# Differences in RNA polymerase II complexes and their interactions with surrounding chromatin on human and cytomegalovirus genomes

**DOI:** 10.1038/s41467-022-29739-x

**Published:** 2022-04-14

**Authors:** Benjamin M. Spector, Mrutyunjaya Parida, Ming Li, Christopher B. Ball, Jeffery L. Meier, Donal S. Luse, David H. Price

**Affiliations:** 1 Department of Biochemistry and Molecular Biology, The University of Iowa, Iowa City, IA 52242 USA; 2 Department of Internal Medicine, The University of Iowa, Iowa City, IA 52242 USA; 3 Department of Epidemiology, The University of Iowa, Iowa City, IA 52242 USA; 4 Veterans Affairs Health Care System, Iowa City, IA 52242 USA; 5 Department of Cardiovascular and Metabolic Sciences, Lerner Research Institute, Cleveland Clinic, Cleveland, OH 44195 USA

**Keywords:** Genome informatics, DNA

## Abstract

Interactions of the RNA polymerase II (Pol II) preinitiation complex (PIC) and paused early elongation complexes with the first downstream (+1) nucleosome are thought to be functionally important. However, current methods are limited for investigating these relationships, both for cellular chromatin and the human cytomegalovirus (HCMV) genome. Digestion with human DNA fragmentation factor (DFF) before immunoprecipitation (DFF-ChIP) precisely revealed both similarities and major differences in PICs driven by TBP on the host genome in comparison with PICs driven by TBP or the viral-specific, late initiation factor UL87 on the viral genome. Host PICs and paused Pol II complexes are frequently found in contact with the +1 nucleosome and paused Pol II can also be found in a complex involved in the initial invasion of the +1 nucleosome. In contrast, viral transcription complexes have very limited nucleosomal interactions, reflecting a relative lack of chromatinization of transcriptionally active regions of HCMV genomes.

## Abstract (web summary)

Here the authors digested chromatin with DNA fragmentation factor (DFF) prior to chromatin immunoprecipitation (DFF-ChIP) to depict transcription complex interactions with neighboring nucleosomes in cells. Applying this method to human cytomegalovirus (HMCV)-infected cells, they find that the viral genome is underchromatinized, leading to fewer transcription complex interactions with nucleosomes.

## Introduction

Regulation of human gene expression is accomplished by a highly orchestrated interplay between the transcription machinery and its chromatinized genomic template. The required general Pol II initiation factors are instructed by Mediator and a host of more specific transcription factors to utilize selected promoters as sites of initiation^1^. However, the default state of the genome is repressive because of global nucleosome deposition, which must be relieved by chromatin remodelers. Assembly of the preinitiation complex (PIC) occurs over sequences surrounding the transcription start site (TSS) and an upstream region depleted of nucleosomes (NDR)^2^. A strongly positioned +1 nucleosome has a boundary around 50 bp downstream from the TSS^3^. On active promoters, this nucleosome and several downstream nucleosomes are often marked by tri-methylation of histone H3 on lysine 4 (H3K4me3)^4,5^. Both PIC assembly and pausing by newly-initiated Pol II have been linked to interactions with the +1 nucleosome^6–10^, but the nature and functional significance of these interactions remain incompletely understood. Existing global methods to visualize and quantify sites of transcription and the locations of the transcriptional machinery within the local chromatin landscape have had limited success in addressing these questions. Occupancy by the +1 nucleosome is fairly uniform across most human promoters while Pol II occupancy is highly variable and generally much lower^6^, resulting in a potentially misleading correlation between two disparate signals. Recently there have been a number of highly informative structural studies of PICs^11–14^, but the abundance of PICs on cellular chromatin has not been adequately determined and their positioning over promoter elements has been primarily inferred from in vitro studies.

The interface of transcription and chromatin on the HCMV genome is even more poorly defined. Lytic infection can propagate only in non-dividing cells. The process begins with delivery of a nucleosome-free viral genome to the nucleus, where the standard host Pol II machinery drives transcription from the major immediate early promoter. Expression of viral immediate early proteins then allows expression of early genes^15,16^. The early genes encode the machinery for viral DNA replication and for transcription of a group of late genes, which requires a special set of viral-specific Pol II initiation factors^17–20^ that likely replace some of the host initiation factors. While ChIP-PCR on individual loci and genome-wide studies have revealed changes in chromatinization throughout the viral lifecycle^21–24^, it is not clear what regions on each of the hundreds of viral genomes present late in infection are occupied by nucleosomes. Nothing is known about how Pol II transcription complexes interface with any chromatin that may be present. Our recent work demonstrated promiscuous transcription initiation from thousands of promoters across the ~240,000 bp dsDNA genome, consistent with the idea that chromatinization of the HCMV genomes during lytic infection is incomplete^25^. Increasing our knowledge of how HCMV transcription is regulated is important for identification of potential therapeutic targets since the virus infects about 60% of the population. HCMV is a significant cause of death in immunocompromised individuals^26^ and a leading viral cause of birth defects^27^.

Our group recently described a nuclear run-off method to directly observe engaged Pol II interacting with the +1 nucleosome^6^. We digested nuclei with the double-stranded endonuclease human DNA fragmentation factor (DFF) and then chased nascent transcripts to the resulting run-off sites. We found that many, but not all, paused polymerases were abutted to the +1 nucleosome. Because DFF digestion preserved the viability of transcription complexes and their relation to chromatin, we decided to investigate whether combining it with chromatin immunoprecipitation would allow improved insight into localization and positioning of transcription complexes. We find that we are able to quantitatively visualize PICs as well as interactions between PICs and paused transcription complexes with the downstream chromatin. We apply the method to primary human foreskin fibroblasts (HFFs) productively infected with HCMV, allowing a direct comparison of the chromatin neighborhoods of human genomic Pol II promoters with their more poorly characterized counterparts on the viral genome.

## Results

Our study began with a characterization of our initial DFF-Seq dataset GSE139237 generated from HeLa cells^6^. Fragments from DFF-Seq were primarily derived from protection of DNA by nucleosomes^6^. To investigate the regions around promoters, active genes in HeLa cells were identified by truQuant^28^ which finds the most highly utilized TSS (MaxTSS) for each expressed gene in this case from HeLa PRO-Seq data^25^. Fragments generated by DFF that were present in a 2000 bp region centered on the MaxTSS of each of the 12,201 promoters were collected and the distribution of fragment lengths was compared to those from the entire DFF-Seq dataset. In the total dataset, peaks of fragment sizes corresponding to mono-, di-, and tri-nucleosome were 163, 326, and 512 bp respectively, while those values were 161, 298 and 452 in the truQuant subset (Supplementary Fig. 1a). This demonstrates that many nucleosomes around promoters are essentially close packed and that this should now be considered a property of human promoter-proximal chromatin structure.

Because MNase has been used extensively to examine chromatin, we wanted to compare properties of DFF to those of MNase. Most MNase digestions are size selected to eliminate over-digestion products, but this was not done during a published time course of MNase digestion of Drosophila nuclei^29^ (GSE128689). One minute of digestion resulted in a range of fragments from about 50 to 400 bp with about half being around the size of mono-nucleosomes (Supplementary Fig. 1b). As the digestion progressed, subnucleosomal fragments predominated (Supplementary Fig. 1b). Critically, DFF digestion of nuclei sufficient to generate primarily mono-nucleosomes resulted in much less invasion of protected nucleosomal DNA relative to MNase digestions carried to comparable levels. To examine any sequence preferences for DFF cleavage, sequences surrounding 520 million cut sites were examined. A slight preference for specific a sequence was seen (Supplementary Fig. 1c) with 6.8% of the sites having AAANT directly on one of the two sides of the cut. Although DFF is a homodimer that mostly cuts DNA to form blunt ends^30^, there was no preference to having this sequence on both sides. In comparison, a similar analysis of MNase digestions shows that MNase has a narrower sequence preference but with greater preference for particular bases around the cut site for both human^31^ (GSE36979) (Supplementary Fig. 1d) and Drosophila nuclei (Supplementary Fig. 1e, f). The actual sequence preference is somewhat different in the datasets compared. G is strongly disfavored +1 of the nick site in human and A is much more favored at that same positon in Drosophila. In both cases, MNase greatly prefers to cut at sites with a + 1 positioned A, but will virtually never cut with a G/C positioned +1 from the cut site. This preference for cleavage results in substantial GC bias in the recovered fragments (positive positions) following MNase digestion. DFF does not lead to any appreciable GC or AT bias in recovered fragments.

### DFF-ChIP reveals differing promoter architecture on human and HCMV genomes

**Fig. 1:**
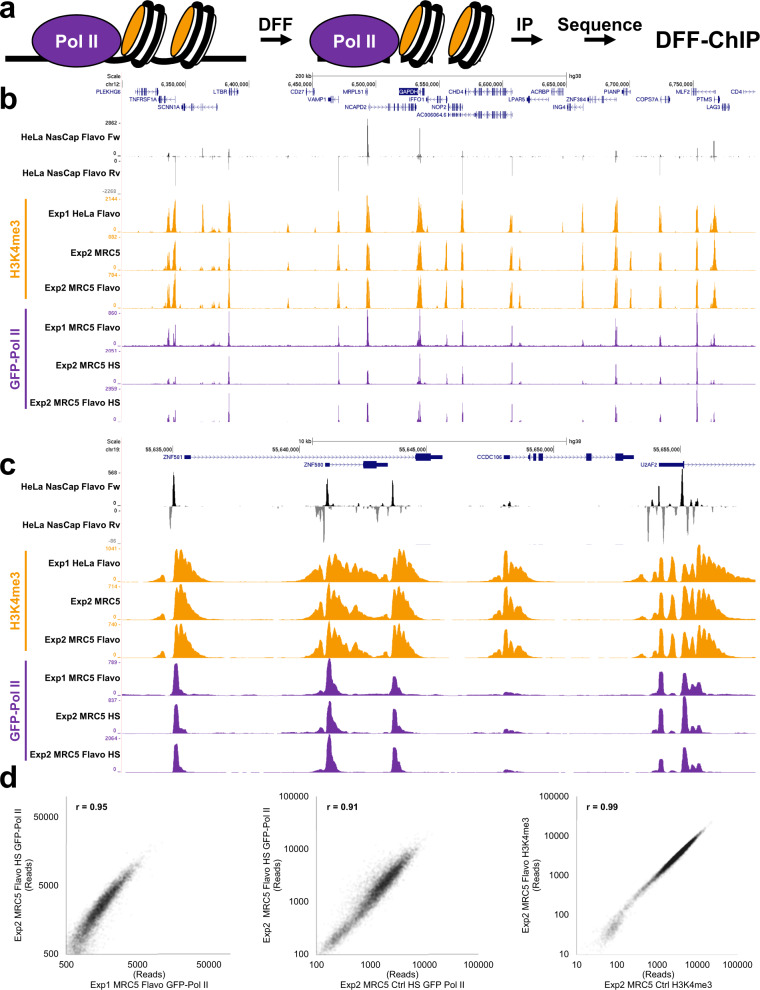
Reproducibility of Pol II and H3K4me3 DFF-ChIP. **a** Diagram of the DFF-ChIP method. Nuclei isolated from uncrosslinked cells in the presence of EDTA are digested with DFF and lightly sonicated to release soluble DNA complexes. The complexes are then immunoprecipitated, library prepped, and sequenced. **b**, **c** Genome browser tracks of Pol II DFF-ChIP (purple) and H3K4me3 DFF-ChIP (orange) from HeLa and MRC5 GFP-Pol II cells generated in two different experiments (Exp1 and Exp2). HS denotes that samples were washed with high salt. Browser tracks of Flavo NasCap PRO-Seq (black/gray) show transcription data. **d** Correlation plots of datasets from Exp1 and Exp2. Reads in 10,000 bp windows centered on the MaxTSS of 12,229 HFF truQuant promoters were summed and plotted against sums from other datasets. Pearson correlations (r) are provided.

Because DFF can generate primarily nucleosome-sized fragments without significant internal cutting, we performed two initial experiments (Exp1 and Exp2) to explore its use for DNA fragmentation for H3K4me3 and Pol II ChIP-Seq. In Exp1, nuclei from non-crosslinked HeLa or MRC5 cells expressing a GFP-tagged Pol II^32^ were digested with DFF for 1 h to generate primarily mono-nucleosomes. The resulting chromatin was immunoprecipitated with antibodies to H3K4me3 modification (HeLa cells) or GFP (MRC5 cells) and the associated DNA was prepared for sequencing (Fig. 1a). For Exp2, the same procedure was performed only using MRC5 cells and under higher salt (HS) washing conditions for GFP-Pol II immunoprecipitation. A table describing the cells, treatments before or after isolation of nuclei, antibodies for the ChIP for of all experiments performed as well as a key as to which datasets provide evidence of reproducibility is provided in the Supplementary Data 1 file (Library Statistics Excel sheet).

The DFF-ChIP results were compared to NasCap PRO-Seq data^6^ from HeLa cells. Paused Pol II is evident from the tall peaks in the PRO-Seq data over a 500,000 bp region of the human genome (Fig. 1b). Peaks of Pol II and H3K4me3 occupancy in the DFF-ChIP tracks exhibited strong visual correlation. Greater detail can be observed across a 30,000 bp region (Fig. 1c). Each promoter exhibits clear nucleosome phasing in the H3K4me3 dataset surrounding a NDR that supports Pol II initiation in both cell types. Additionally, Pol II DFF-ChIP signal overlaps with the PRO-Seq signal but also extends slightly further downstream. This downstream signal likely results from Pol II that is abutted to the +1 nucleosome such that DFF cannot cleave between polymerase and the nucleosome^6^. The complex H3K4me3 and Pol II patterning on the majority of promoters is well replicated even across cell types and growth conditions between Exp1 and Exp2 indicating the robustness of the DFF-ChIP method (Fig. 1c). To further validate the method’s reproducibility, the sum of reads found in a 10 kb window centered on each of 12,201 truQuant MaxTSSs from different experiments were directly compared and strongly correlated between the datasets (Fig. 1d).

**Fig. 2:**
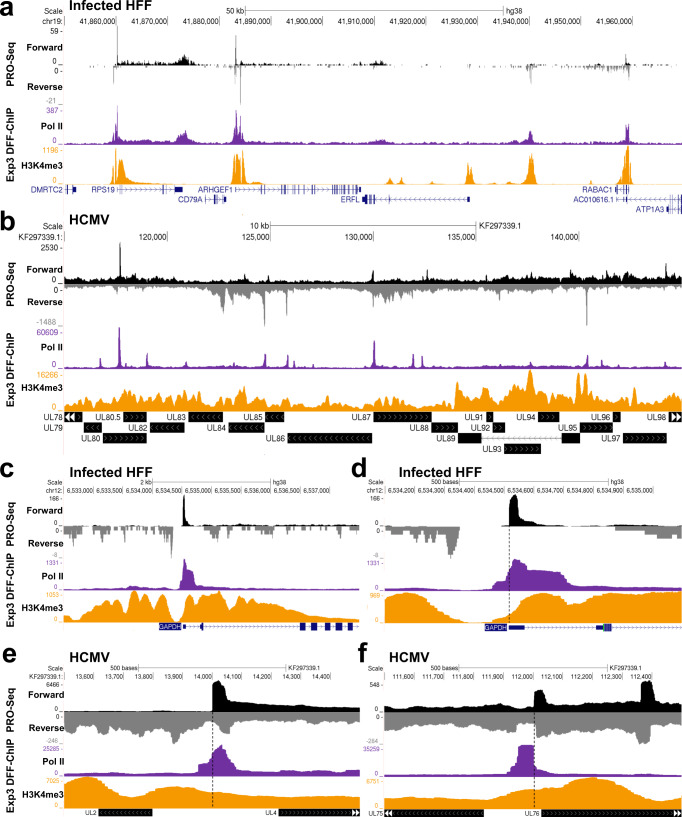
Representative genome browser tracks of H3K4me3 and Pol II DFF-ChIP from Infected HFFs. **a**, **b** Genome browser tracks of PRO-Seq, Pol II DFF-ChIP, and H3K4me3 DFF-ChIP from HFFs 48 hpi (Exp3) on the hg38 and TB40/E genomes. **c**, **d** Genome browser tracks of PRO-Seq, Pol II DFF-ChIP, and H3K4me3 DFF-ChIP (Exp3) showing the GAPDH promoter in 5000 and 1000 bp windows. A dotted line denotes the MaxTSS. Genome browser tracks of PRO-Seq, Pol II DFF-ChIP, and H3K4me3 DFF-ChIP (Exp 3) of the TB40/E genome showing an early (**e**) and a late (**f**) promoter in 1000 bp windows. A dotted line denotes the MaxTSS.

Given the success of these initial experiments, DFF-ChIP was then applied to contact inhibited HFFs infected with HCMV (TB40/E) for 48 h (Exp3, see Supplementary Data 1 file) to shed light on the chromatin environment around viral promoters. DFF-ChIP results were compared to PRO-Seq data from similarly infected HFFs for broad regions across the host (Fig. 2a) and viral genomes (Fig. 2b). As expected on the host genome, Pol II and H3K4me3 corresponded with paused Pol II evident from the PRO-Seq data. The viral genome was pervasively transcribed as previously demonstrated^25^ and Pol II DFF-ChIP correlated with the PRO-Seq signal when transcription of both strands is taken into account. Unlike the host genome where H3K4me3 signal is found only around promoter regions, the entirety of the HCMV genome is covered, at levels ranging from relatively low to more enriched irrespective of Pol II occupancy (Fig. 2b).

Additional insight was obtained when the results from individual promoters on the host and viral genomes were examined. The host GAPDH promoter features nucleosome phasing around a NDR that supports Pol II initiation (Figs. 2c, d). In contrast, the viral early gene promoter for UL4 and late gene promoter for UL76 show no obvious phasing of H3K4me3 modified nucleosomes (Fig. 2e, f). Critically, the two viral promoters are not found in NDRs. Because initiation cannot take place when the promoter is occluded by a nucleosome^33,34^, the results suggest that some of the nucleosomes detected are present only on regions of viral genomes that are not transcribed. The Pol II DFF-ChIP signal on GAPDH overlaps with the PRO-Seq signal and has abutted Pol II signal (Fig. 2d). However, additional protections extending farther upstream are also apparent. Neither viral promoter shows evidence of nucleosome-abutted Pol II and signal from the paused Pol II not abutted to the +1 nucleosome (free) predominates for the HCMV early promoter (Fig. 2e), but is not the main signal for the late promoter (Fig. 2f). The location of upstream protection seen in all three promoters suggests the presence of Pol II in preinitiation complexes which are undetectable by prior ChIP methods, and surprisingly, those complexes predominate for the viral late promoter (Fig. 2f).

### Transcription complexes and their interactions with nearby nucleosomes can be visualized using fragMaps

**Fig. 3:**
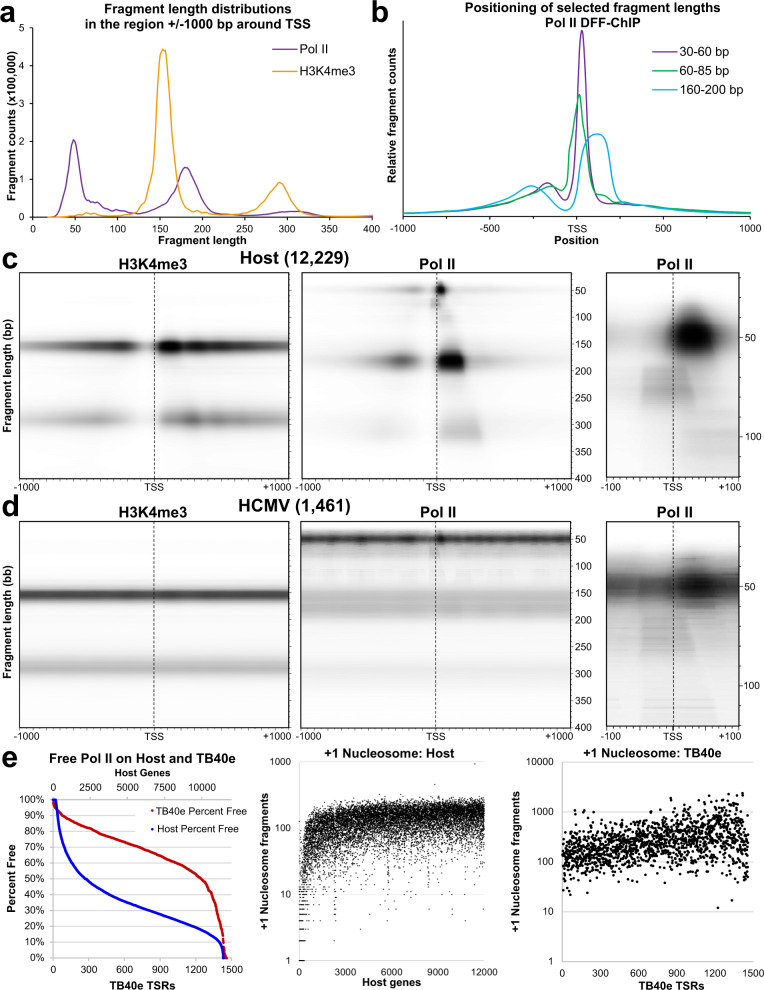
Visualizing and quantifying transcription complexes and chromatin utilizing fragMaps. **a** Length distribution of fragments contained in the region ±1000 bp of the MaxTSS of 12,229 HFF truQuant promoters for Pol II and H3K4me3 DFF-ChIP datasets (Exp4). **b** Fragment count of selected ranges of fragments from the Pol II DFF-ChIP (Exp4) positioned across the 2000 bp region centered on the MaxTSS of 12,229 HFF truQuant promoters. The three curves were normalized by making the area under the curves equal. **c** fragMaps of H3K4me3 and Pol II (Exp4) for the 12,229 HFF truQuant promoters showing 18–400 bp fragments that are positioned ±1000 bp around the MaxTSS. A zoomed fragMap showing 18–120 bp fragments that are positioned ±100 bp around the MaxTSS is also shown (right). **d** fragMaps of H3K4me3 and Pol II (Exp4) for the 1461 TB40/E TSRs found on the HCMV genome showing fragments from 18–400 bp that are positioned ±1000 bp around the MaxTSS. A zoomed fragMap showing 18–120 bp fragments that are positioned ±100 bp around the MaxTSS is also shown (right). The resulting H3K4me3 HCMV fragMap was lightened to aid in visualization. **e** Quantification of percentage of the free Pol II feature signal relative to total paused Pol II (free + abutted) on both the host and TB40/E genomes using Exp4 data on a promoter by promoter basis sorted from highest to lowest free Pol II (Left). Fragment count of the Nuc1 feature from the H3K4me3 dataset (Exp4) on host (middle) and TB40/E (right) genomes sorted by percentage of the free Pol II feature relative to total paused Pol II (free + abutted) on a promoter by promoter basis from highest to lowest free Pol II as is shown in the graph on the left.

Because the patterns of DFF protection around promoters suggested that DFF-ChIP could separate engaged and initiating transcription complexes, fragment length analysis was explored as a means to analytically separate them. To facilitate this, a fourth experiment was performed on HCMV infected HFFs (48 hpi) using a larger set of antibodies for ChIP that could differentiate these complexes (Exp4, see Supplementary Data 1 file). Comparison of DFF-ChIP for H3K4me3 and Pol II across specific regions on the host and viral genomes for Exp3 and Exp4 clearly demonstrates high reproducibility of the patterns of occupancy (Supplementary Fig. 2a, b). In addition, a number of strong correlations between Exp3 and Exp4 were found when signals around host and viral promoters were compared (Supplementary Fig. 2c, d). A set of 12,229 truQuant MaxTSSs derived from HFF PRO-Cap data was utilized and the distribution of fragment sizes within a 2000 bp window centered on these MaxTSSs from the Pol II and H3K4me3 Exp4 datasets revealed several distinctive groups of fragment sizes (Fig. 3a). A majority of H3K4me3 fragments in this window center on 158 bp in length and a smaller population were about 294 bp in length, corresponding to mono- and close packed di-nucleosomes. In the Pol II dataset, two abundant fragment sizes at ~50 bp and 180 bp were the most prevalent, with a smaller population of fragment sizes around 75 bp. Fragment ranges corresponding to each of the three most common Pol II populations in the 2000 bp windows were then chosen and positioned relative to TSSs (Fig. 3b). The number of reads in each of these fragment size ranges was normalized to total reads in the 2000 bp window to emphasize the protected footprints of the less abundant ~75 bp fragments. Both the ~50 bp and ~180 bp fragments align slightly downstream of the TSS in the pause region while the ~75 bp fragments span the TSS, as would be expected for PICs. The positioning of the ~50 bp and the ~180 bp fragments are consistent with free paused Pol II and Pol II abutted to the +1 nucleosome. The same analysis was performed utilizing infected HFF Pol II and H3K4me3 DFF-ChIP data from Exp3, which gave similar results (Supplementary Fig. 3a, b).

Since metaplots limit how many fragment lengths can be shown together in a meaningful and accurate way, we created a method that more holistically depicts the distribution of all fragments around TSSs. The output of this visualization method simultaneously captures the size, amount, and position of each fragment across all promoters analyzed in an easily viewable fragMap. Host HFF fragMaps were generated utilizing the 12,229 TSSs found with truQuant and TSSs on the HCMV genome were found utilizing transcription start regions (TSRs) identified using TSR-finder on a PRO-Cap dataset generated from HCMV infected HFFs that resulted in 1461 non-overlapping 200 bp regions centered on a MaxTSS^25^. For each fragment length, the coverage at each position ±1000 bp around all found TSSs was calculated and averaged. These averages for each fragment length at each position were then stacked with the shortest fragments on top and longest on the bottom. Fragment lengths included in this view span from 18 to 400 bp. More focused fragMaps were also created ±100 bp around the MaxTSS using 18–120 bp fragments. For most fragMaps, black values (overall darkness of the image) are set by the maximum average read value in the window. Because the black value in each fragMap is influenced by the recovery of the IP, absolute amounts of visible complexes should only be compared within the same fragMap.

FragMaps for the Pol II and H3K4me3 Exp4 datasets generated a snapshot of transcription complexes and chromatin features on the host and HCMV genomes. Host H3K4me3 fragMaps show very well positioned nucleosomes relative to the TSS and a clear NDR (Fig. 3c). This patterning of H3K4me3 marked nucleosomes closely resembles fragMaps generated using the DFF-Seq HeLa truQuant dataset, except that the signal becomes fainter as the distance from the TSS increases (Supplementary Fig. 3c). Pol II fragMaps of the host genome show free paused Pol II (~50 bp fragments downstream from the TSS), Pol II positioned over the TSS (~75 bp fragments), Pol II abutted to the +1 nucleosome (~180 bp fragments downstream of the TSS), and Pol II associated with the first two nucleosomes (~320 bp fragments downstream of the TSS) (Fig. 3c). More detailed criteria for features are provided in the Supplementary Data 1 file (Feature Description sheet). The size of the fragments bearing Pol II associated with the first two nucleosomes supports the finding that nucleosomes around promoters are more closely spaced than in bulk chromatin (Supplementary Fig. 1a). A complex of ~100 bp, located downstream of the TSS, is also visible in the Pol II fragMaps. Possible origins for this unanticipated complex will be discussed later. Divergent transcription occurs at variable distances upstream of the sense TSS on the host genome, so the upstream region displays a similar, but less well-defined pattern compared to transcription in the sense direction. Pol II and H3K4me3 fragMaps from Exp 3 datasets displayed all the same features and were virtually indistinguishable from those using Exp4 datasets (Supplementary Fig. 3d) demonstrating that the method is robust and reproducible.

Major differences in the patterns of H3K4me3 and Pol II on the host fragMaps were found on the HCMV fragMaps. The HCMV H3K4me3 signal was not positioned around the majority of promoters (Fig. 3d). HCMV Pol II fragMaps confirm the pervasiveness of Pol II transcription with Pol II visible across the fragMap (Fig. 3d). However, this pattern is also a result of fragMaps being a summation of 200 bp TSRs centered in a 2000 bp window. Should another TSR be within 2000 bp of the centered TSR it will contribute additional signal offset from the TSS. This causes a light background of particularly sized fragments across the entire visualized region. The fragments near the TSS show free paused Pol II, Pol II positioned over the TSS in ~75 bp fragments, and an additional complex of ~50 bp positioned just upstream of the TSS that is not present on host fragMaps. This ~50 bp protection over the TSS is especially evident in Exp3 viral fragMaps (Supplementary Fig. 3e). Nucleosome-abutted Pol II, which is the most abundant feature seen on the host genome, is present but at a vastly lower level than free Pol II on the HCMV genome.

The apparent differences in Pol II association with the +1 nucleosome on the host and HCMV genomes prompted us to quantify how frequently Pol II encounters an immediately downstream nucleosome in these two cases. The number of reads in features corresponding to the free Pol II, abutted Pol II, and +1 nucleosome based on genomic position of fragment centers and fragment size were quantified from Pol II and H3K4me3 datasets (Supplementary Data 1 file). Analysis of free and abutted Pol II demonstrated that 36% of the total engaged Pol II signal arises from free Pol II on the host genome. In contrast to the host, 74% of the engaged Pol II was free on the HCMV genome. On a promoter by promoter basis, 85% have more free than abutted Pol II signal on the HCMV genome, whereas only 18% do on host (Fig. 3e). Examination of the fragment count of +1 nucleosome-sized fragments from the H3K4me3 dataset shows that the host promoters with the absolute highest percentage of free Pol II have little H3K4me3 modified +1 nucleosome, but most promoters have a similar amount of it (Fig. 3e). Although the H3K4me3 signal over the viral genome has a similar value in terms of reads, there are about a hundred times more viral genomes and thus the H3K4me3 modified nucleosome occupancy over the viral genomes is about 1% of that in the host.

### Detection and characterization of TBP-driven PICs and UL87-driven viral PICs

**Fig. 4:**
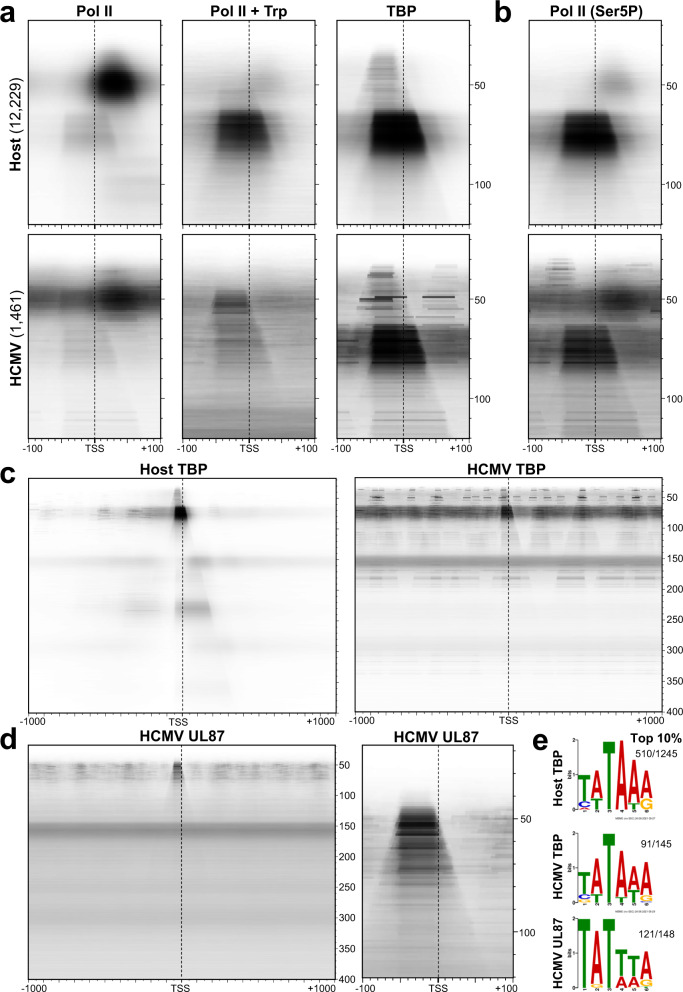
Detection and characterization of TBP-driven PICs and UL87-driven viral PICs. **a**, **b** fragMaps of Pol II, Pol II + triptolide (Trp), TBP, and Ser5P datasets (Exp4) for the 12,229 truQuant HFF promoters showing 18–120 bp fragments positioned ±100 bp around the MaxTSS. HCMV (TB40/E) fragMaps were generated from 1461 TB40/E TSRs. A dotted line denotes the TSS. **c** fragMaps of TBP (Exp4) for the 12,229 truQuant showing 18–400 bp fragments positioned ±1000 bp around the MaxTSS. The HCMV (TB40/E) fragMap was generated from 1461 TB40/E TSRs. **d** UL87 fragMaps (Exp4) were generated from 1456 Towne TSRs. Fragment lengths and positions depicted are the same as in **a** and **b**. A dotted line denotes the TSS. **e** Logos generated with MEME Suite 5.3.1 from the top 10% promoters/TSRs with the most fragments present in the TBP PIC or UL87 PIC feature as detected by DFF-ChIP. Parameters were: ZOOPS, search only given strand, 1 motif, 6 bp motif. Fractions represent the number of sequences matching the sequence motif out of the number of input sequences. E values for the three Logos were: TBP host, 1.5e−398; TBP HCMV, 3.6e−038; UL87 HCMV, 1.4e−082.

The existence of Pol II-containing complexes that extended upstream of the MaxTSS strongly suggested that these features correspond to PICs, so Pol II DFF-ChIP was performed after treatment of cells for an hour with 1 µM triptolide (Exp4), an inhibitor of transcription initiation, that should increase the PIC relative to paused Pol II. That is exactly what was found for the ~75 bp feature on the host and viral genomes (Fig. 4a). Triptolide treatment also more clearly revealed the ~50 bp feature directly upstream of the TSS, which is unique to the viral genome (Fig. 4a). As an additional verification that ~75 bp protections arose from uninitiated Pol II, Exp1 and Exp2 DFF-ChIP were reanalyzed since they utilized different wash conditions in a GFP-Pol II tagged MRC5 cell line^32^ and GFP nanobody beads. The immunoprecipitations in these experiments were carried out with 150 mM or 1 M salt wash conditions. The ~75 bp feature was preferentially lost during the high salt conditions as expected^35^ for uninitiated Pol II (Supplementary Fig. 4a). DFF-ChIP was then performed targeting the TATA-binding protein (TBP), a critical component of the host PIC. FragMaps generated from the TBP dataset show primarily the ~75 bp feature (Fig. 4a), indicating that these complexes are indeed TBP-containing PICs. Additionally, TBP fragMaps reveal protections that share the relatively sharp upstream edge with the full TBP PIC but are much smaller in size, about 40 bp. These likely result from TBP-containing complexes prior to incorporation of Pol II and assembly of the complete PIC. DFF-ChIP was also performed utilizing a Pol II antibody that targets the serine 5 phosphorylation on the CTD of Pol II (Ser5P). Ser5P modification is carried out by CDK7, a component of the initiation factor TFIIH^36^. The resulting fragMaps demonstrate that Ser5P antibodies recognize modified Pol II in the TBP PIC on both the host and viral genome. However, the ~50 bp PICs on the viral genome were not detected (Fig. 4b) suggesting that the Pol II in those complexes is not phosphorylated by CDK7. There is a difference in the relative amounts of PIC and free paused Pol II detected with the Pol II antibody and the Ser5P antibody. The epitope on the Pol II large subunit recognized by the Pol II antibody is located deep within the PIC^13^ and potentially masked by TFIIE and TFIIH, while the Ser5P epitope would be difficult to mask because of its repetitive occurrence and disordered structure. Therefore, we favor the idea that the Ser5P results are more representative of Pol II levels in all TBP-driven promoter-proximal complexes. Supporting this idea, when MRC5 cells containing a GFP-tagged Pol II were immunoprecipitated with GFP nanobodies, the PIC was a major species (Supplementary Fig. 4a).

Ser5P ChIP with MNase digestion without fragment size selection has recently been carried out in mouse fibroblasts^37^ (GSE142179). To determine if DFF has advantages over MNase, we compared the library fragment size distributions and fragMaps generated from our data to those from the mouse cells. Although the overall range in fragment sizes was about the same, individual complex classes were much more clearly delineated in the DFF generated fragments (Supplementary Fig. 4b). Critically, transcription complexes that were prominent in the DFF-ChIP data were not detected by MNase-ChIP (Supplementary Fig. 4b). This important result demonstrates a clear advantage of using DFF instead of MNase for analysis of transcription complexes and the surrounding chromatin.

Large TBP fragMaps provided further insight into the chromatin differences between the host and HCMV genomes (Fig. 4c). Some TBP associated fragments share an upstream edge with the PIC but are ~230 bp in length which is consistent with an interaction between the PIC and a nucleosome. These interactions are mostly absent on the HCMV genome presumably because the viral genome is less chromatinized. Significant amounts of the host PICs driving divergent transcription also connect with their downstream nucleosome. As with the free-standing PIC, the PIC/+1 nucleosome feature on the host genome was salt sensitive (Supplementary Fig. 4c). Previous analysis of downstream sequences relative to TSSs revealed periodic elements that likely serve to position the +1 nucleosome^6^, suggesting a connection between the TSS and nucleosome positioning. Considering that both genic-oriented and divergent PICs are normally associated with well-positioned immediately-adjacent nucleosomes, it is further likely that the PIC, TSS, and +1 nucleosome connection is important for specifying and/or facilitating transcription initiation.

Unlike the early HCMV transcriptional program that relies entirely on the host general Pol II transcription machinery, beta- and gamma herpesviruses have unique late promoters containing a TATT upstream element that recruits virally-encoded late transcription factors^38,39^. Since only one of the two distinct Pol II-containing PICs on the viral genome corresponds to the host complex driven by TBP, we posited that the ~50 bp virus-specific PIC is based on UL87, one of the viral late transcription factors which associates with the TATT element^17^. DFF-ChIP was performed with a Towne strain of HCMV expressing a HA-tagged UL87 (Exp4). FragMaps demonstrated that the ~50 bp viral PICs were almost exclusively recovered with the HA antibody (Fig. 4d). Interestingly, unlike the 75 bp TBP-PICs the 50 bp UL87-PICs did not cover the TSS.

In an attempt to correlate upstream sequence elements with the extent of PIC assembly, UL87 PIC and TBP PIC features at each truQuant promoter on the host or on each TSR of the HCMV genome were quantified (Supplementary Data 1 file). The size and position of these features were selected such that no overlap was allowed between them. Each region was then rank ordered by the amount of the UL87 PIC feature or the TBP PIC feature. Logos were generated by MEME analysis^40^ from the −38 to −19 region for the PICs in the top decile of occupancy on the host and viral genomes (Fig. 4e). Such analysis recapitulated the expected TA-rich binding motifs with TBP preferring sequences containing TATA and UL87 preferring sequences containing TATT. UL87 evidently has a stricter requirement for TATT since MEME discovered the UL87 logo in 121 of 148 sequences queried (81%), while MEME discovered the TBP logo in and 91 of 145 viral sequences (63%) queried and 510 of 1245 host sequences (41%).

### TBP and UL87 are functionally distinct but not mutually exclusive on HCMV promoters

**Fig. 5:**
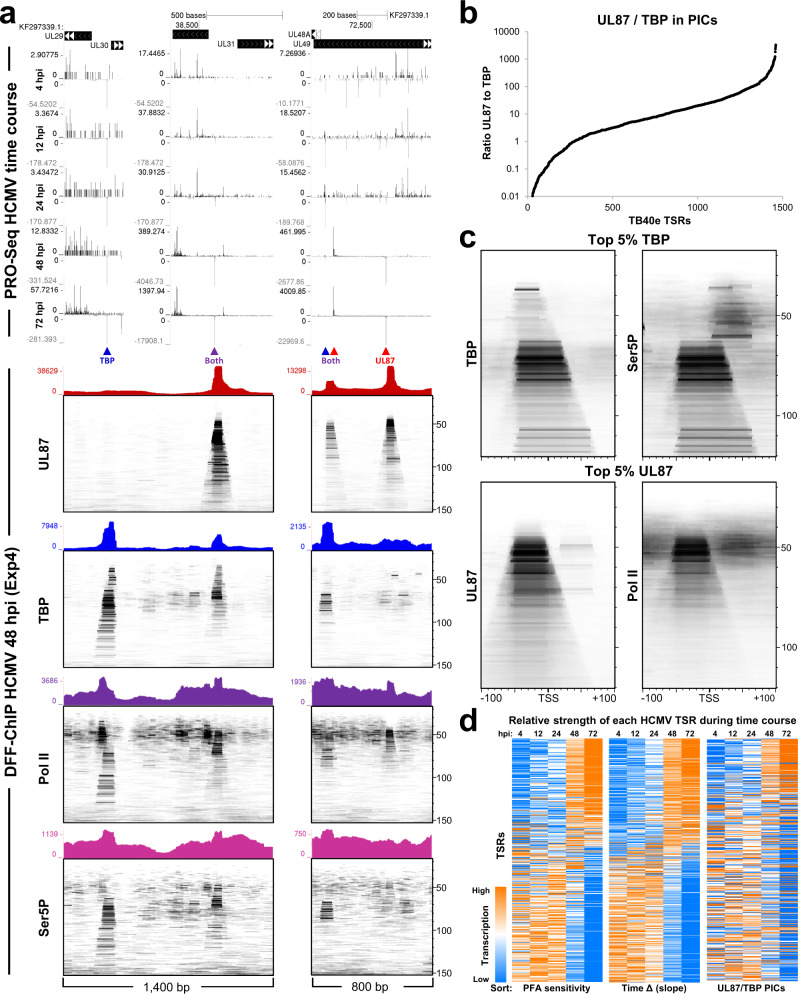
Timing of promoter usage during HCMV infection is related to the ratio of UL87 and TBP PICs. **a** PRO-Seq tracks depicting 5′ ends of reads from HCMV infection time course including 4, 12, 24, 48 and 72 hpi datasets in 1400 and 800 bp regions of the viral genome. Below are corresponding DFF-ChIP tracks from Exp4 and fragMaps of the same region. **b** Quantification of the relative usage of TBP and UL87. The amount of TBP PIC feature counted from the TBP dataset was normalized to the amount of UL87 PIC feature counted from the UL87 dataset for all 1461 TB40/ETSRs (Exp4). The ratio of UL87 PIC to TBP PIC was used to sort the TSRs by TBP PIC dominance and then plotted. **c** FragMaps for the top 5% TBP and UL87 dominated (**b**) TSRs utilizing 18–120 bp sized fragments positioned ±100 bp around the TSSs. Top TBP TSRs are depicted using TBP and Ser5P (Exp4) datasets whereas UL87 TSRs are depicted using UL87 and Pol II (Exp4) datasets. **d** A set of 795 TSRs with greater than 100 MaxTSS 5′ ends (±5 bp) when all time points are summed were selected and sorted based on PFA sensitivity, slope, or UL87/TBP. Each TSR had each time point value normalized to library size and each TSR was colored independently. The time point with the highest relative transcription was colored orange and lowest colored blue.

To characterize the transcription of the HCMV genome throughout lytic infection and its relation to UL87 and TBP usage, PRO-Seq was carried out at multiple different time points of infection and compared to 48 hpi DFF-ChIP data. Time points include two early times (4 and 12 hpi), 24 hpi which is around the beginning of replication, and two late times (48 and 72 hpi) in which high levels of viral replication have occurred and during which UL87 function is critical. Representative regions of the HCMV genome containing early and late genes are depicted in a 1400 bp region and an 800 bp region with corresponding HCMV genomic fragMaps below showing fragments from the UL87, TBP, Pol II, and Ser5P Exp4 datasets (Fig. 5a). The early promoter UL29 has only TBP, Pol II, and Ser5P fragments associated with it (Fig. 5a, blue TBP arrow), while the late intragenic promoter in UL49 only has UL87 and Pol II fragments (Fig. 5a, red UL87 arrow). TBP and UL87 driven promoters can occur very close to each other (Fig. 5a, red and blue Both arrows) or can even drive transcription from the exact same TSS (Fig. 5a, purple Both arrow). Overlapping PICs such as these would be expected to compete for occupancy on the HCMV genome. Quantification of 5′ end reads found in an 11 bp window around the MaxTSS of these promoters shows that TBP-driven TSSs are more active early in comparison to UL87 PICs that are active late (Supplementary Fig. 5a). Because some promoters are driven by both TBP and UL87 the distinction between early versus late gene transcription is more complex than a simple separation of TBP and UL87 driven promoters.

Nearly all promoters show some level of UL87 and TBP usage and to determine to what extent promoters are shared, the UL87/TBP PIC ratio was calculated for all 1461 TSRs and plotted (Fig. 5b). The results indicate that the relative levels of UL87 and TBP PICs vary by many orders of magnitude across the 1461 TSRs. Therefore, to make comparisons between TBP and UL87 PICs only the top and bottom 5% of TSRs sorted by PIC ratio were utilized to prevent contaminating signal from the other class of PIC. To compare initiation efficiency, fragMaps were generated utilizing the TBP and Ser5P datasets for the top TBP TSRs and UL87 and Pol II datasets for the top UL87 TSRs (Fig. 5c). As noted above, the Pol II antibody epitope in the large subunit of Pol II is significantly masked in TBP PICs^13^, so the Ser5P Pol II signal was used to visualize Pol II in TBP driven TSRs. The strong correlation of PIC feature counts from the Ser5P dataset in comparison to TBP and Pol II datasets also advocates for this approach (Supplementary Fig. 5b). These fragMaps reveal that UL87 and TBP driven TSRs yield similar amounts of engaged Pol II in relation to PIC amounts. This result was initially surprising given the prominence of UL87 PIC peaks in comparison to detected paused Pol II downstream of those PICs, which seemed to indicate poor UL87 initiation. However, it is possible that a significant fraction of ~50 bp UL87 features may not have Pol II associated with them and this is supported by the relative lack of protection downstream of the TSS. Critically, on both promoter classes the PIC is as prominent on chromatin as paused Pol II indicating that the PIC is far more prevalent on the genome than expected.

To quantify how TBP and UL87 usage is related to early or late expression, transcription from each promoter was quantified by counting 5′ ends in an 11 bp window centered on the MaxTSS of each TSR at each time point. The fractional usage for each promoter at each time point was displayed in a heatmap after normalizing to the total number HCMV reads in each time point. 795 of the 1461 promoters that each had a total of at least 100 reads across the time course were then sorted based on reliance on DNA replication (PFA sensitivity)^28^, on a slope calculated from the 5 time points for each promoter or the ratio of the associated UL87 to TBP PICs (Fig. 5d). Each promoter was colorized individually based on the relative usage across the time course. The three sorts gave similar patterns of promoters with early (orange to blue) and late (blue to orange) transcription kinetics. Each TSR’s PFA sensitivity was then plotted against slope to classify how TSRs with primarily TBP (blue) and UL87 (red) PICs behaved in relation to time of expression (Supplementary Fig. 5c). The relative preference for TBP or UL87 PICs was calculated as the ratio of TBP/UL87 PICs after normalization of total counts in each feature. This plot shows that UL87 primarily functions on promoters with late transcription kinetics and that many of these promoters are also stimulated by DNA replication. However, and significantly, many promoters with late kinetics are primarily TBP driven showing that TBP also plays a significant role in transcription of some late promoters. The UL22A promoter has a TBP/UL87 ratio of 0.93 and displays late kinetics. There are two main TSSs at all time points that are separated by 3 bp with a shift in the relative usage at early and late time points that is reverted when viral replication is blocked by PFA treatment (Supplementary Fig. 5d). This promoter exemplifies a clear competition for formation of TBP and UL87 PICs.

### Nucleosomes on the HCMV genome are irregularly spaced

**Fig. 6:**
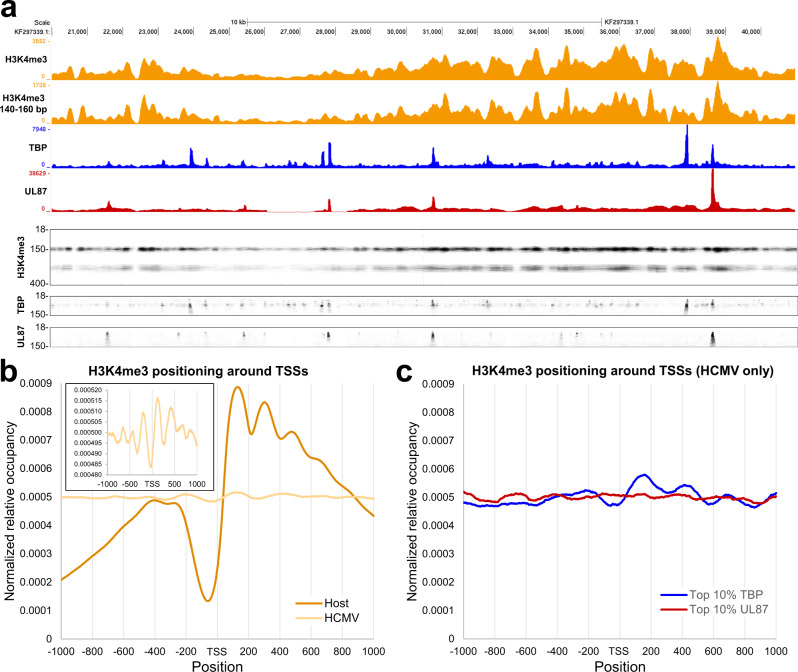
Analysis of TBP and UL87 PICs on the HCMV genome. **a** Genome browser tracks from all fragment sizes for H3K4me3, TBP, and UL87 datasets and for the 140–160 bp fragments from H3K4me3 (all Exp4) compared directly to fragMaps of the same region of the HCMV genome. The H3K4me3 fragMap depict fragments between 18–400 bp whereas TBP and UL87 fragMaps depict fragments between 18–150 bp. **b** Normalized metaplot of H3K4me3 signal around the MaxTSS of 12,229 HFF truQuant promoters and 1461 TB40/E TSRs. The inner graph shows the HCMV metaplot with a different Y-axis (Exp4). **c** Normalized metaplot of H3K4me3 signal around the top 10% of HCMV TSRs determined by amount of TBP PIC feature or UL87 PIC feature (Exp4).

To directly assess if sites of initiation correlated with H3K4me3 modified nucleosomes, genome browser tracks and genomic fragMaps for TBP, UL87 and H3K4me3 were compared. There is little evidence for a correlation of sites of transcription initiation and H3K4me3 modification regardless of PIC type (Fig. 6a). This comparison does allow for a rough estimation of the number of nucleosomes that span the 21,000 bp region shown, since ~70–80 H3K4me3 modified nucleosomes are distinguishable. This indicates that on average a nucleosome is positioned every 250–300 bp on the HCMV genome. This average spacing is consistent across the entire HCMV genome (Supplementary Fig. 6). It is unclear what drives nucleosome positioning on HCMV DNA but given the scarcity of transcription-coupled nucleosomes it is unlikely to be related to transcription. Metaplot analysis of all 1461 viral TSRs shows that H3K4me3 modified nucleosomes around promoters are spaced ~250–300 bp in contrast a much more compact spacing around promoters on the host genome of about 150 bp (Fig. 6b). A PIC containing TBP is capable of associating with the +1 nucleosome on the host (Fig. 4c) and this prompted us to investigate if viral TSRs with strong TBP signal have a better positioned downstream nucleosome. Selecting the top 10% of TSRs with the highest level of TBP PICs showed that indeed these TSRs have a slightly stronger +1 nucleosome signal bearing a H3K4me3 modification (Fig. 6c). The same analysis performed with the top UL87 TSRs failed to reveal any clear H3K4me3 marked nucleosome patterning (Fig. 6c). These findings suggest that while TBP PICs may aid in the positioning of a +1 nucleosome, this is not a driving force for nucleosome positioning on the HCMV genome.

### Increasing the extent of DFF digestion reveals a general robustness of features and captures Pol II association with sub-nucleosomal fragments

**Fig. 7:**
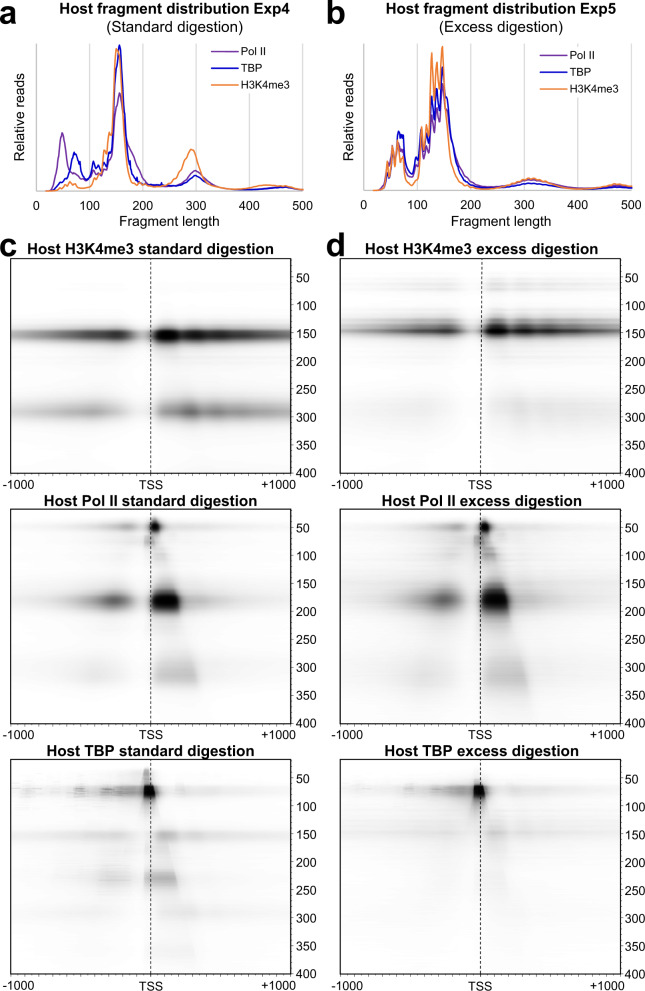
FragMaps resulting from different extents of DFF digestion. **a**, **b** Total fragment distributions from Pol II, TBP, and H3K4me3 datasets from the standard digestion condition (Exp4) and the excess digestion condition (Exp5). **c**, **d** fragMaps of Pol II, H3K4me3, and TBP showing 18–400 bp fragments positioned ±1000 bp around the MaxTSS of 12,229 truQuant promoters with either (**c**) standard (Exp4) or (**d**) excess digestion (Exp5). Exp5 datasets are each a combination of two replicas (*n* = 2).

To confirm reproducibility and determine the effects of more extensive DFF digestion, five datasets were generated in duplicate using HFFs infected with the Towne strain of HCMV (Exp5). The datasets correlated highly within Exp5 and between Exp5 and Exp4 (Supplementary Fig. 7a). Total library fragment length distributions for Pol II, TBP, and H3K4me3 were compared between the initial and more extensively digested experiments (Fig. 7a, b). It was apparent that higher levels of digestion resulted in a division of some complexes into subgroups typically separated by a 10 bp periodicity. Over-digestion did not change the ratio of the free to abutted Pol II (Fig. 7c, d), but the group of Pol II-containing fragments of ~90–110 bp became more apparent after increased digestion. Connections of the TBP PIC to downstream nucleosomes were mostly lost with over-digestion.

The H3K4me3 fragment distributions in Fig. 7 clearly show that DFF can cleave within nucleosomes at higher digestion levels. Two classes of products shorter than the expected protection from the full nucleosome were observed: fragments which apparently resulted from removal of 10 or 20 bp from the nucleosome ends and another population of centered fragments less than 80 bp consistent with protection by the H3/H4 tetramer. The lack of fragments between the two populations suggests that complete loss of the flanking H2A/H2B dimers on the edges of the nucleosome occurs before the central H3/H4 is invaded by DFF. All sub-nucleosomal fragments were centered including those less than 80 bp, further demonstrating that they arose from the H3/H4 tetramer (Supplementary Fig. 7c, left).

To better understand the interaction of Pol II with the +1 nucleosome we specifically considered well positioned nucleosomes downstream of the most focused promoters^6^ (Supplementary Fig. 7b). At such promoters, the upstream edge of the +1 nucleosome is positioned on average at +47, just downstream of the average paused Pol II position at +41. Pol II protects ~20 bp of DNA downstream of the active site^41^, suggesting that the leading edge of the abutted Pol II intrudes ~1.5 DNA turns into the nucleosome. Pol II at this location would disrupt the H3 contact at nucleosome entry but could leave the H2A/H2B contacts with DNA at least partially intact. If invasion by Pol II distorts the nucleosome, this could in turn reveal a cutting site for DFF downstream of the H2A/H2B dimer. DFF cleavage in such a complex would give rise to the 87–107 bp fragment set that we observe in Pol II IPs, more prominently upon over-digestion (Fig. 7, and Supplementary Fig. 7). Other possible origins for this ~100 bp Pol II fragment set can be envisioned, which will be addressed in the Discussion.

## Discussion

Our results provide a deeper understanding of transcription complexes and their interactions with chromatin on both the host and HCMV genomes. Critically, analysis of the length and position of fragments recovered by DFF-ChIP using fragMaps not only provided detailed footprints of PICs and paused Pol II but also directly revealed the interactions of those complexes with the +1 nucleosome. Using HCMV infected HFFs, we demonstrated that Pol II encounters a vastly different chromatin environment on the viral genome than it does on the host genome. Furthermore, our direct visualization of both TBP and UL87-driven PICs sheds light on sequence preferences, dimensions, and shared usage at the two promoter classes.

Our results provide a perspective into the transcriptional processes during lytic infection in relation to the chromatin structure of the HCMV genome. Earlier work suggested that during productive infection, viral genomes form irregular nucleosome arrays^21,42,43^. However, a more recent genome-wide analysis utilizing MNase suggested that the HCMV genome is largely packaged into nucleosomes throughout the viral cycle^24^. Our data confirm that H3K4me3 marked nucleosomes are deposited across the viral genome at low level of occupancy, but we also show in multiple ways that paused Pol II rarely encounters a nucleosome on HCMV DNA. The TBP, Pol II, Ser5P, and UL87 DFF-ChIP signals corresponding to initiating Pol II or PICs often reside in the middle of apparently nucleosome rich regions on HCMV DNA despite the known ability of a nucleosome to block initiation^33,34^. Therefore, those regions of HCMV genomes with nucleosomes over TSSs cannot be transcriptionally active. Transcription complexes themselves report on local nucleosomes by conferring a larger protection footprint when they are in close proximity to a nucleosome. HCMV chromatin very rarely confers these protection patterns. Although it is likely that the large majority of HCMV promoters do not feature modified nucleosomes immediately downstream, it is not possible to prove that this is the case at every individual viral promoter. Post-translationally modified nucleosomes may be positioned on individual loci at some stages of the viral life cycle^22,23^. Overall, we conclude that during lytic infection the HCMV genome is transcribed in a predominantly nonchromatinized state.

The unique ability to recover PICs with DFF-ChIP without prior crosslinking was likely allowed by the use of EDTA during very rapid nuclei isolation^44^ prior to digestion and immunoprecipitation steps. EDTA was included to halt transcription but it may also increase PIC retention by eliminating the destabilization caused by ATP^35^ in abortive initiation and XPB function. Substantial recovery of PICs in DFF-ChIP has allowed a better understanding of their properties in the nucleus including documenting that TBP- and UL87-driven PICs are major features on the host (TBP) and viral genomes (TBP and UL87), equaling or surpassing paused Pol II in amount. The formation of the TBP-containing PIC requires Pol II in a hypophosphorylated state, but after PIC assembly in vitro, phosphorylation of the CTD may occur even prior to formation of the first phosphodiester bond^45,46^. Our data demonstrate that this actually occurs in cells, showing that Ser5P is present on TBP PICs (Fig. 3b). Additionally, we show that a substantial fraction of PICs directly interacts with the +1 nucleosome.

DFF-ChIP also uncovered differences between TBP and UL87 PICs and their interplay on the HCMV genome. TBP PIC footprints correspond to the known in vitro footprint of TFIID, from roughly 40 bp upstream of the TSS to 35 bp downstream^6^. Published human TFIID and PIC structures indicate that TFIID and XPB both contact DNA well downstream of the TSS^2,47^. In contrast, UL87 PIC footprints are only located upstream of the TSS, suggesting that UL87 PICs lack the subunits of TFIID that contact downstream DNA and, crucially, at least the XPB subunit of TFIIH. Since UL87 PICs also lack Ser5P modification, it seems likely that the UL87 PICs we detect lack TFIIH. However, initiation at UL87 PICs is sensitive to inhibition of XPB by triptolide. We therefore propose that while TFIIH is required for UL87 initiation, it is not a stable component of the UL87 PIC that we detect. A recent study regarding ORF24, a UL87 homolog in Kaposi’s sarcoma associated virus^20^, showed association with the Pol II CTD only in the hypophosphorylated state, which is in agreement with our data demonstrating that the Pol II in UL87 PIC is predominantly unphosphorylated^48^. Regardless of these functional differences, both PICs function on a large shared subset of HCMV promoters.

We expected from our earlier study that paused Pol II complexes would be located upstream of the +1 nucleosome or abutted to that nucleosome^6^. Those complexes are evident from DFF-ChIP (Fig. 2c) but high levels of DFF digestion in particular revealed unanticipated Pol II-containing fragments of 87–107 bp that extend downstream into the region that is expected to be protected by the proximal H2A/H2B dimer of the +1 nucleosome (Supplementary Fig. 4d). We suggested above that polymerase invasion of the nucleosome could reveal a site for DFF cleavage downstream of the dimer. However, other recent work suggests a plausible alternative explanation. It was reported that the Chd1 chromatin remodeler associates with +1 nucleosomes, specifically on the promoter-proximal face^49^. Subsequent structural studies showed that in a Chd1-nucleosome complex, a Chd1 domain displaces DNA from the nucleosome surface normally occupied by H2A/H2B^50^. Thus, the 87–107 bp Pol II-containing fragments we detected could have arisen from Pol II paused at the entry of a +1 nucleosome already occupied by Chd1. Presumably the interface of Chd1 and the nucleosome is more easily accessible at high levels of DFF digestion. In the earlier studies it was speculated that displacement of the Chd1 domain at nucleosome entry by the advancing polymerase would activate the remainder of Chd1 to drive displacement of the nucleosome and thus facilitate traversal by Pol II. Thus, this model predicts that once Pol II has displaced the proximal Chd1 domain, full traversal of the +1 nucleosome should be efficient^50^. This is consistent with the fact that we did not detect Pol II-containing complexes by DFF-ChIP corresponding to pausing just upstream of H3/H4 tetramer of the +1 nucleosome, as might have been predicted from earlier in vitro studies^51^. Other results based on micrococcal nuclease digestion patterns indicated that in Drosophila +1 nucleosomes frequently lack the proximal H2A/H2B dimer^52^. However, as just noted, we do not have evidence from our experiments for a Pol II barrier at the H3/H4 tetramer of the +1 nucleosome.

Evidence presented here and elsewhere^6^ suggests that DFF offers significant advantages over MNase in detecting and quantifying transcription complexes and chromatin features. Because DFF lacks RNase, unlike MNase, it can be combined with global methods that examine nascent transcripts. Likely because DFF is a dsDNase it does not easily invade nucleosomes or other complexes that the nicking activity of MNase does. This allows visualization of transcription complexes that have not been seen with techniques using MNase. Future usage of DFF including DFF-ChIP holds great potential in uncovering intricacies of chromatin architecture, like the finding here that nucleosomes are close packed in promoter-proximal regions. Targeted immunoprecipitation of various modified histones or histone replacements are but a few of the possibilities for these investigations. For direct inquiries into transcription, further DFF-ChIP experiments that target the general transcription factors involved in initiation, pausing, and productive elongation are of great interest and will aid in uncovering the ways that transcription complexes interact with nucleosomes. In addition, we expect DFF-ChIP to be applicable to the targeted investigation of many more specific chromatin associated factors. Finally, it is possible that DFF might be a useful reagent in examination of 3D organization of the genome.

## Methods

### Viruses and cells

HCMV TB40/E BAC4 and Towne UL87HA were used in this study. The construction and use of the Towne UL87-HA recombinant virus was described previously^28^. Primary human foreskin fibroblasts were isolated from de-identified, discarded newborn infant foreskins. The University of Iowa IRB (IRB ID# 201702734) determined that the use of de-identified, discarded human tissues/cells is not considered human subject research.

### Infections and treatments

HFFs were maintained in Minimum Essential Medium (Gibco, 11095080) supplemented with 5% fetal bovine serum (Gibco, 26140079) and 1% penicillin-streptomycin (Gibco, 15140122). Confluent (contact inhibited) HFF monolayers in T-150 cm^2^ flasks were used for these studies. The culture medium was refreshed 24 h prior to infection. On the day of infection, all but 12 mL of the conditioned medium was removed and set aside. The remaining 12 mL of medium was inoculated with HCMV at a multiplicity of infection of 3 infectious units per cell (MOI of 3). Viral adsorption was carried out for 90 min. The medium containing viral inoculum was then replaced with 12 mL of the conditioned medium. For experiments involving treatment with flavopiridol (Flavo; final concentration, 1 μM), or triptolide (final concentration, 1 μM), 6 mL of conditioned medium was temporarily removed 1 h before cells were harvested. This medium was treated with 6 μL of 2 mM Flavo (NIH AIDS Reagent Program 9925z) in DMSO, 6 μL of 2 mM triptolide (Sigma, T3652) in DMSO or 6 μL of DMSO alone. Once inoculated with drug, the 6 mL of medium was immediately returned to the flask for a final 12 mL of culture medium. At 48 h post-infection, cells were lysed and cell nuclei were isolated and held in frozen storage until use, as described previously^28^.

### DFF-ChIP Seq

DFF was purified as previously described^6^. For Exp4 ~6 million nuclei from human foreskin fibroblasts infected for 48 h with human cytomegalovirus strains TB40/E or a mutant Towne virus expressing UL87-HA were digested with ~15 µg of DFF in 20 mM HEPES (pH 7.6), 5 mM magnesium acetate, 100 mM potassium acetate, 5 mM DTT, for 1 h at 37 °C. Digestions were carried out in batch where possible for all nuclei of the same treatments conserving the ratio of nuclei to DFF. Digestion was halted with the addition EDTA to a concentration four times that of magnesium and nuclei were subsequently split for individual IPs. Nuclei were lightly sonicated for 20 s at 40% amplitude using Qsonica Q800R3 Sonicator and the supernatant was collected and brought up to 1 mL with solution containing 10 mM Tris (pH 7.5), 100 mM sodium chloride, 1 mM EDTA, and TritonX-100 such that the final concentration was 0.1%. The supernatants were precleared for 20 min over Protein A (Sigma P9424) or G Sepharose (Sigma P3296) beads. Afterwards, the supernatants were removed from beads and immunoprecipitated with ~2.5 µg of antibodies for Pol II (Santa Cruz, sc-55492), TBP (Abcam, ab51841), Ser5P (3E8 mouse monoclonal obtained from D. Eick, LMU), H3K4me3 (Abcam, ab8580), or 10 µL HA-tag (Cell Signaling Technology, C29F4), and overnight at 4 °C with rotation. Next, samples were incubated with Protein A beads (Protein G beads for Ser5P IPs) for 2 h at 4 °C with rotation. The beads were than washed five times with 10 mM Tris (pH 7.5), 150 mM sodium chloride, 1 mM EDTA, and 0.1% TritonX-100 for five minutes per wash. Bound material was than eluted twice with 50 µL of 10 mM Tris (pH 7.5), 1% SDS, and 1 mM EDTA incubated at 65 °C for 5 min. Eluted material was subsequently treated with 20 µg RNAse A for 30 min at 37 °C and then 40 µg of Proteinase K for 2 h at 65 °C. The same protocol was used for the all DFF-ChIP experiments with slight modifications. For MRC-5 expressing GFP-Pol II and HeLa cell lines in Exp1 and Exp2, 12 million nuclei were digested with 30 µg DFF. After splitting and preclearing on Protein A sepharose beads, GFP-Pol II samples were incubated with Chromotek GFP-Trap beads for 4 h, washed five times with either the same buffer as above or one containing 1 M sodium chloride for ~1 min each, and eluted as described above. Samples from HeLa cells were immunoprecipitated, eluted, and treated the same as Exp4 except with ~1 min washes. Exp3 was performed exactly as Exp4 was done, but again with ~1 min washes. For Exp5 utilizing HFFs infected with Towne, 3 million HFF cells infected with Towne UL87-HA were incubated with ~25 µg DFF. All libraries were cleaned up utilizing Invitrogen PureLink PCR purification kit. All libraries were prepared using the KAPA Hyper Prep Kit according to their protocol (Roche 7962312001). For libraries in experiments 1 and 2 we utilized Illumina TruSeq adapters. For all subsequent libraries, we created custom adapters utilizing the TruSeq sequences that contained an 8 bp UMI immediately downstream of the index. Test amplifications were performed on each library to determine the number of cycles necessary to obtain enough library material for sequencing. Full-scale amplification was then performed, fragments were quantified using an Agilent Bioanalyzer 2100, and then libraries were pooled and size selected from 135-1000 bp using a BluePippin. Prior to submission, proper size selection was confirmed with reanalysis using the Agilent Bioanalyzer. Libraries were sequenced on either an Illumina HiSeq 4000 or a NovaSeq 6000 and converted to fastq using bcl2fastq2 (version 2.2) by the Iowa Institute of Human Genetics.

### PRO-Seq and PRO-Cap

PRO-Seq and PRO-Cap libraries were prepared as previously described^28^, with a few modifications. Frozen nuclei isolated from HFF infected with HCMV TB40/E (MOI 3) for 48 or 72 h were thawed on ice, gently pelleted, and resuspended in 40 μL of a nuclear run-on buffer (20 mM HEPES (pH 7.6), 5 mM magnesium chloride, 100 mM potassium chloride, 5 mM dithiothreitol (DTT), and 0.6 U/μL SUPERase-In (Invitrogen AM2696)). Approximately 100,000 moth Sf21 nuclei were spiked into HFF nuclei prior to pelleting. Nuclei were warmed to 37 °C and then combined with 20 μL of a 3X nuclear run-on mix (20 mM HEPES (pH 7.6), 5 mM magnesium chloride, 100 mM potassium chloride, 5 mM DTT, 1.5% Sarkosyl, and 60 uM biotinylated ATP, UTP, GTP, and CTP (Perkin Elmer NEL544, NEL543, NEL545, and NEL542, respectively)). Samples were pulse-vortexed after the addition of nuclear run-on mix and incubated at 37 °C for 10 min. Reactions were quenched with 40 μL of 50 mM ethylenediaminetetraacetic acid (EDTA) and 300 μL of Trizol LS (Ambion 10296028), and total RNA was extracted according to manufacturer protocol. All subsequent steps in PRO-Seq library preparation were carried out as previously described^28^. PRO-Cap library preparation followed a similar procedure, except excluded the RNA hydrolysis step and included RNA polyphosphatase and terminator exonuclease treatments to ensure the integrity of nascent RNA 5’ end capture. A detailed description of the PRO-Cap protocol has been published^25^. Reverse-transcribed libraries were PCR amplified for 11 (PRO-Seq) or 17 (PRO-Cap) cycles, subjected to analysis on an Agilent Bioanalyzer 2100, pooled in equimolar ratios, size-selected on a Sage Science Blue Pippen (BDF2010 cassette, 135–600 bp fragments selected), and sequenced on an Ilumina HiSeq 4000 with 150 bp paired-end reads at the University of Iowa Genomics Division. Raw data were trimmed, mapped, deduplicated, and processed into tracks as previously described^28^.

### DFF-ChIP analysis

Initial workup of data was performed using DNAfastqtoBigWig (https://github.com/P-TEFb/DNAfastqtoBigWig). This is a linux based, multi-thread capable, Next Generation Sequencing (NGS) data analysis program with a command line interface. It performs the standard NGS data processing steps including, downloading sequencing data from a given web server, trimming adapter sequences from the sequencing data, aligning the trimmed data to a given list of genomes, generating mapping statistics for the aligned data, deduplication of aligned fragments using their Unique Molecular Identifiers (UMI), and finally, generating tracks for each sample in bigwig format. The program automatically accomplishes the following steps. Raw sequences in Fastq format were downloaded from the Iowa Institute of Human Genetics (IIHG) Genome Sequencing web server using wget command. Next, adapter sequences were trimmed from these sequences using trim_galore 0.6 (https://github.com/FelixKrueger/TrimGalore/releases/tag/0.6.6) while retaining only paired end trimmed sequences of at least 18 bp in size. These sequences were aligned with UCSC hg38, and Genbank TB40/E and Towne HCMV assemblies using bowtie v1.2.2 to generate alignments in sam format. UMIs reads were used to deduplicate the aligned reads which were then converted into bed files. Unstranded tracks were generated for each sample by first converting bed into bedGraph format using bedtools v2.26, and subsequently into bigwig format using the Kent UCSC utility program called bedGraphToBigWig. All datasets are described in the Supplementary Data 1 file. Raw and processed sequencing data can be obtained from GEO GSE185763.

### Fragment distribution plots

Fragment size frequencies were calculated by counting the number of times a single fragment size or a range of fragment sizes are present in a given sample or overlap to a list of genomic intervals of a specific size. Bedtools v2.26 intersect program was used to generate overlap data between fragment and genomic intervals. Bash and awk scripts were used to generate counts for a single or a range of fragment sizes. Next, fragment sizes and their associated counts were sorted from short to long order. Additionally, fragment size counts were normalized to generate their relative amounts by dividing them with the total number of fragments present in a sample. MS Excel was used to plot relative and absolute counts of fragment sizes for each sample using scatter with straight lines option.

### Average base distribution plots

The number of times a nucleotide is present at each position within a specified genomic interval was calculated as follows. Bedtools v2.26 getfasta program was used to generate FASTA files while maintaining same strand orientation for ±100 bp genomic intervals centered on fragment starts and ends. For these analyses all intervals centered on fragment starts were marked positive strand and intervals centered on fragment ends were marked negative strand. A custom linux based, multi-thread capable python script called ABD.py (https://github.com/P-TEFb/ABD) was run to generate absolute counts for each nucleotide across the given genomic intervals. Absolute nucleotide counts were converted to fractions by dividing them with the total number of sequences. MS Excel was used to plot base fractions across the genomic interval using scatter with straight lines option. Finally, the following color code was used for these plots: A was blue (hex code #2222ff), T was orange (hex code #ff6600), G was gray (hex code #bbbbbb), and C was yellow (hex code #dddd00).

### MaxTSSs analysis

The truQuant program^28^ was run on the PRO-Cap data after applying a modified blocklist of Pol I and III transcription units from GenecodeV27 (Supplementary Data 1 file – hg38 blocklist sheet) GSE113394^25^ generated from uninfected HFF cells and DMSO bound NasCap data GSE139237^6^ generated from HeLa cells using published parameters to generate host specific MaxTSSs. 12,229 (PRO-Cap) and 12,201 (NasCap) MaxTSSs associated with known host genes were used for further analysis. TsrFinderM2^6^ was run on PRO-Cap datasets from TB40/E infected HFFs 72 hpi GSE185763 and Towne infected HFFs 96 hpi GSE113394 using published parameters to generate HMCV specific MaxTSSs. MaxTSSs associated with RNA 4.9 and without a ±1000 bp genomic sequence were excluded from further analysis. 1461 TB40/E and 1456 Towne MaxTSSs were used for further analysis.

### Feature analysis

Feature analysis was performed by only counting fragments of a specific length whose center lay within a specific genomic interval using Bedtools v2.26 intersect program and simple awk commands for each MaxTSSs in the host and HMCV (TB40/E and Towne) genomes. The feature parameters used for both full fragment and fragment center analyses are provided in the Supplementary Data 1 file. The features consisted of fragments 30–65 bp with a fragment center between +10 and +45 for free Pol II, 140–205 bp with a fragment center between +65 and +140 for abutted Pol II, 120–175 with a fragment center between +65 and +175 for +1 nucleosomes, 64–88 with a fragment center between −18 and −2 for TBP PICs, and 40–63 with a fragment center between −36 and −18 for UL87 PICs. The engaged Pol II feature was found by summing the free Pol II and abutted Pol II counts. Quantification of percentage of Pol II that was either abutted or free was done by selecting promoters with at least one read in either the abutted or free features and that have a MaxTSS of at least 10. Percentage of free Pol II was found relative to engaged Pol II for the whole dataset as well as a gene by gene basis. Quantification of PIC amounts to engaged Pol II was done by first normalizing the TBP PIC fragments from the TBP dataset and UL87 PIC fragments from the UL87 dataset. The ratio between these normalized values was used to rank order promoters by UL87 or TBP dominance and then the ratio of PIC to engaged Pol II was calculated. The Ser5P dataset was utilized for analysis of TBP PICs.

### FragMaps

Heatmaps displaying the average distribution and position of fragments from DFF-Seq and DFF-ChIP across individual genome intervals or collections of intervals were created using fragMap.py (https://github.com/P-TEFb/fragMap). In general, collections were centered on the MaxTSSs from truQuant annotations that provide the most highly utilized TSS for each active gene with proper strand orientation. In some cases, subsets of the truQuant annotations were used. The data was generated by counting the total number of fragments of each fragment size between 18 and 400 bp across the genomic interval. The aspect ratio, number of pixels, intensities assigned, and the shape of major and minor tick marks were controlled. The desired aspect ratio was implemented by choosing a discrete number of pixels for each base or fragment size. The values at each horizontal position of the fragment sizes were used to assign intensities using the gray.colors function in R with 0 being white and maximum being black. A linear relationship between relative read value and intensity was utilized. Black was set at the maximum read value for most frag maps. To correct for human inaccuracies in perception of dark and light patterns on heatmaps, a gamma correction of 0.5 was applied to all fragMaps.

### Statistics

Pearson correlation coefficient “r” was calculated to demonstrate the reproducibility of our datasets using the MS excel CORREL function. Pearson’s r computes the effect of change in one variable compared to the change in another variable. MEME motif discovery tool was used to short sequence motifs in a set of longer DNA sequences. Motifs with an E-value less than 0.05 were considered as significantly enriched in our dataset. E-value is an estimation of seeing similar motifs of identical width and contributing sites in a similar size dataset of random sequences.

### Reporting summary

Further information on research design is available in the Nature Research Reporting Summary linked to this article.

## Supplementary information

Supplemental Figures

Editorial Assessment Report

List of supplementary information

Supplementary Data 1

Reporting Summary

## Data Availability

The data that support this study are available from the corresponding author upon reasonable request. Raw and processed sequencing data generated for this manuscript can be obtained from GEO (GSE185763) without restriction. Previously published PRO-Cap datasets of uninfected HFFs and 96 hpi Towne infected HFFs (GSE113394), HeLa NasCap data (GSE139237), Drosophila MNase-Seq data (GSE128689), Human MNase-Seq data (GSE36979), and Drosophila MNase-ChIP data (GSE142170) are available from GEO without restriction. Source data are provided with this paper.

## Code availability

The following custom scripts are available: DNAfastqtoBigWig (https://github.com/P-TEFb/DNAfastqtoBigWig), trim_galore 0.6 (https://github.com/FelixKrueger/TrimGalore/releases/tag/0.6.6), ABD.py (https://github.com/P-TEFb/ABD), fragMap.py (https://github.com/P-TEFb/fragMap), truQuant (https://github.com/meierjl/truQuant).
