# Supplementary material for: Differences in RNA polymerase II complexes and their interactions with surrounding chromatin on human and cytomegalovirus genomes
Source: Nat Commun. 2022 Apr 14;13:2006. doi: 10.1038/s41467-022-29739-x (PMC9010409; doi:10.1038/s41467-022-29739-x)
Supplement: Supplementary file 1 — Supplemental Figures [file 41467_2022_29739_MOESM1_ESM.pdf]

Differences in RNA polymerase II complexes and their interactions with surrounding chromatin on human and cytomegalovirus genomes

Benjamin M. Spector[a], Mrutyunjaya Parida[a], Ming Li[a,b,c,d], Christopher B. Ball[a], Jeffery L. Meier[b,c,d], Donal S. Luse[e], and David H. Price[a]

[a]Department of Biochemistry and Molecular Biology, The University of Iowa, Iowa City, IA 52242, USA

[b]Department of Internal Medicine, The University of Iowa, Iowa City, IA 52242, USA

[c]Department of Epidemiology, The University of Iowa, Iowa City, IA 52242, USA

[d]Veterans Affairs Health Care System, Iowa City, IA 52242, USA

[e]Department of Cardiovascular and Metabolic Sciences, Lerner Research Institute, Cleveland Clinic, Cleveland, OH, 44195, USA.

Keywords: RNA polymerase II, preinitiation complexes, chromatin, human cytomegalovirus, TBP, UL87, DFF ChIP-Seq

Supplemental Figures

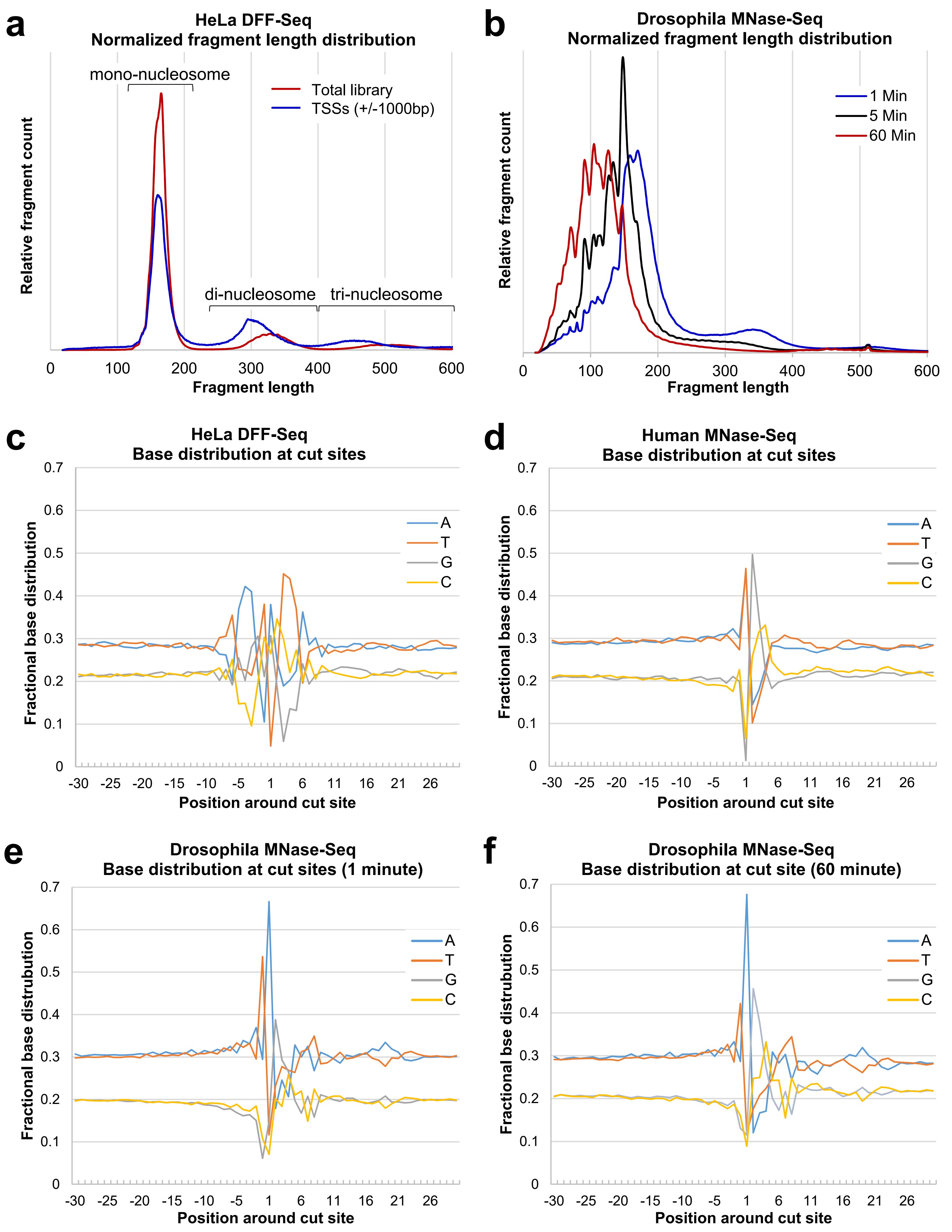
a
HeLa DFF-Seq
Normalized fragment length distribution
mono-nucleosome
di-nucleosome
tri-nucleosome
Total library
TSSs (+/-1000bp)
Relative fragment count
Fragment length
b
Drosophila MNase-Seq
Normalized fragment length distribution
1 Min
5 Min
60 Min
Relative fragment count
Fragment length
c
HeLa DFF-Seq
Base distribution at cut sites
Fractional base distribution
Position around cut site
d
Human MNase-Seq
Base distribution at cut sites
Fractional base distribution
Position around cut site
e
Drosophila MNase-Seq
Base distribution at cut sites (1 minute)
Fractional base distrubution
Position around cut site
f
Drosophila MNase-Seq
Base distribution at cut site (60 minute)
Fractional bse distrubution
Position around cut site
A
T
G
C

Supplementary Fig. 1. Analysis of DFF-Seq and comparison of DFF-Seq to MNase-Seq
a Fragment length distribution for the total DFF-Seq library[6] and for fragments +/- 1000 bp from the MaxTSS of 12,201 truQuant HeLa promoters. Fragment counts were normalized. Mono-, di-, and tri-nucleosome are demarcated. **b** Fragment length distribution of MNase-Seq data generated in Drosophila utilizing 1 min, 5 min, and 60 min time points of digestion[51]. The three curves were normalized by making the area under curves equal. **c** Base distribution of the surrounding 60 bp around the 5′ ends DFF-Seq fragments. **d** Base distribution of the surrounding 60 bp around the 5′ ends of MNase-Seq fragments from human cells[30]. **e,f** Base distribution of the surrounding 60 bp around the 5′ ends of MNase-Seq fragments generated from the 1 minute and 60-minute time point of digestion in Drosophila[51].

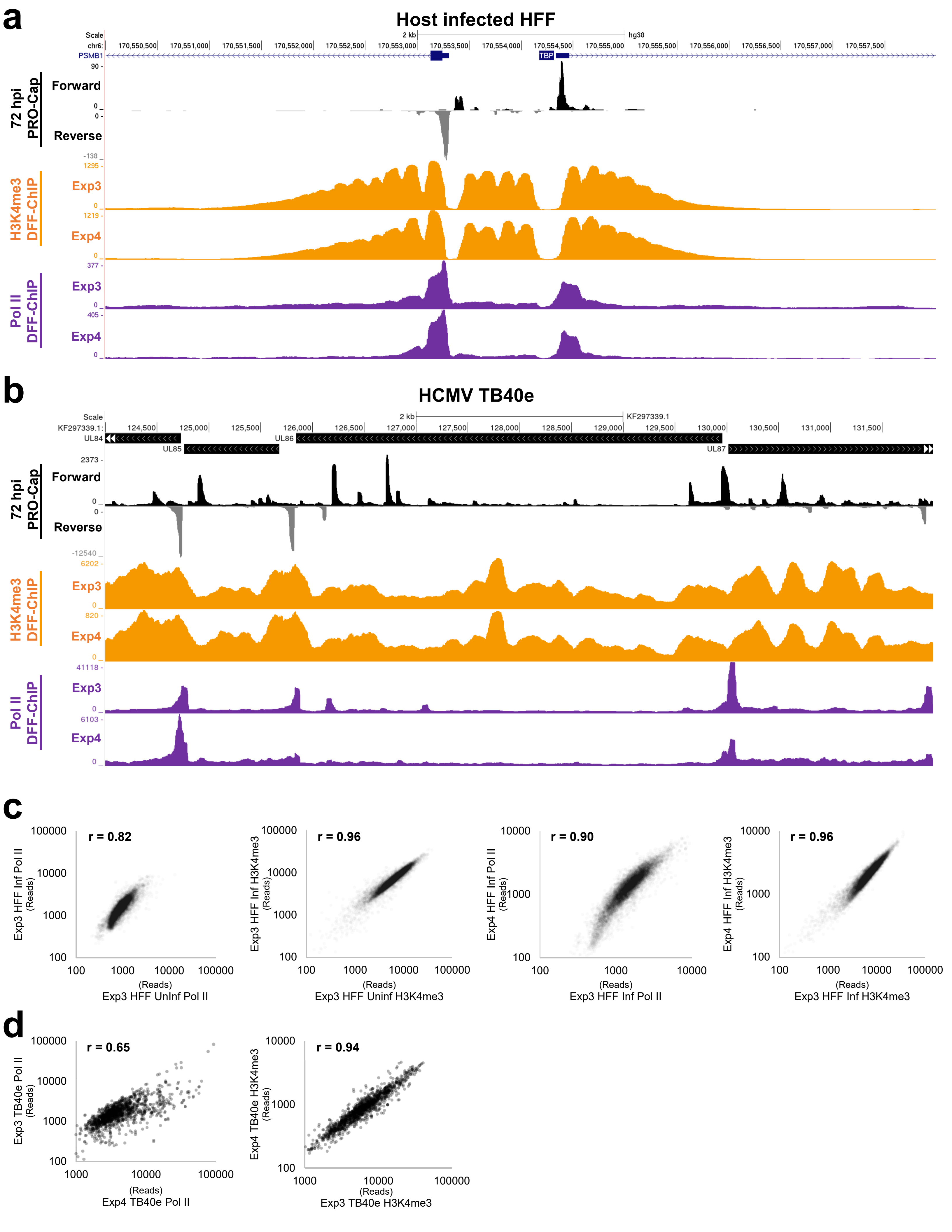
a
Host infected HFF
Scale
2 kb
hg38
chr6:
170,550,500
170,551,000
170,551,500
170,552,000
170,552,500
170,553,000
170,553,500
170,554,000
170,554,500
170,555,000
170,555,500
170,556,000
170,556,500
170,557,000
170,557,500
PSMB1
TBP
72 hpi PRO-Cap
Forward
Reverse
90
0
-138
H3K4me3 DFF-ChIP
Exp3
Exp4
1295
1219
Pol II DFF-ChIP
377
405
b
HCMV TB40e
Scale
2 kb
KF297339.1
KF297339.1:
124,500
125,000
125,500
126,000
126,500
127,000
127,500
128,000
128,500
129,000
129,500
130,000
130,500
131,000
131,500
UL84
UL85
UL86
UL87
2373
-12540
6202
820
41118
6103
c
r = 0.82
Exp3 HFF Inf Pol II (Reads)
Exp3 HFF UnInf Pol II (Reads)
r = 0.96
Exp3 HFF Inf H3K4me3 (Reads)
Exp3 HFF Uninf H3K4me3 (Reads)
r = 0.90
Exp4 HFF Inf Pol II (Reads)
Exp3 HFF Inf Pol II (Reads)
r = 0.96
Exp4 HFF Inf H3K4me3 (Reads)
Exp3 HFF Inf H3K4me3 (Reads)
100
1000
10000
100000
d
r = 0.65
Exp3 TB40e Pol II (Reads)
Exp4 TB40e Pol II (Reads)
r = 0.94
Exp4 TB40e H3K4me3 (Reads)
Exp3 TB40e H3K4me3 (Reads)

Supplementary Fig. 2. Reproducibility of DFF-ChIP on infected HFFs
a, b Genome browser tracks DFF-ChIP H3K4me3 and Pol II tracks from Exp3 and Exp4 and 72 hpi PRO-Cap transcription data. Representative 8000 bp regions are shown on the host and HCMV genomes. **c,d** Correlations between Exp3 and Exp4 datasets. Host reads in 10,000 bp windows centered on the MaxTSS of 12,229 HFF truQuant were summed and plotted against sums from other datasets (**c**). Viral reads from 1,461 200 bp transcription start regions (TSRs)[25] identified using TSR-finder were summed and plotted against sums from other datasets (**d**). Pearson correlations (r) are provided.

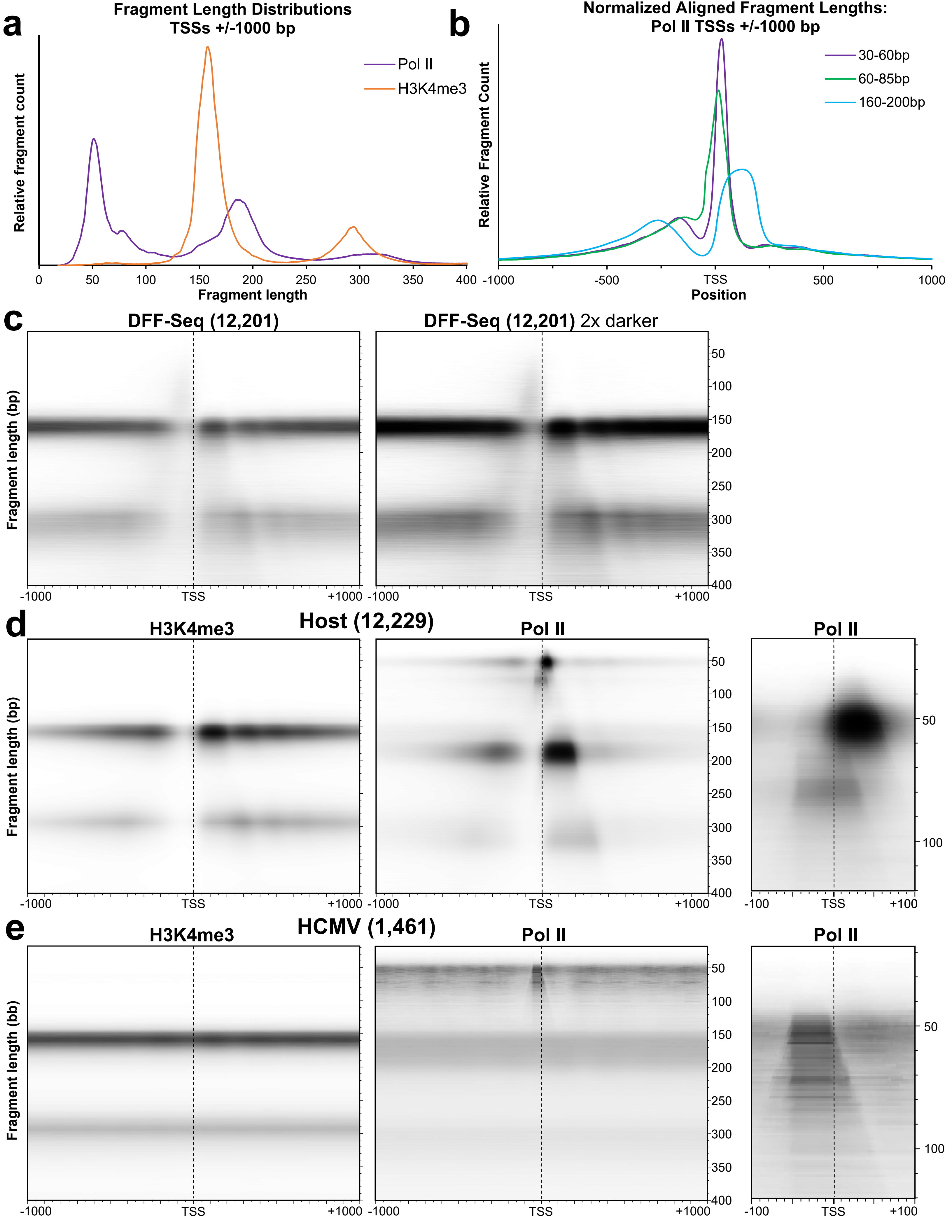
a
Fragment Length Distributions
TSSs +/-1000 bp
Relative fragment count
Pol II
H3K4me3
0 50 100 150 200 250 300 350 400
Fragment length
b
Normalized Aligned Fragment Lengths:
Pol II TSSs +/-1000 bp
Relative Fragment Count
30-60bp
60-85bp
160-200bp
-1000 -500 TSS 500 1000
Position
c
DFF-Seq (12,201)
DFF-Seq (12,201) 2x darker
Fragment length (bp)
50 100 150 200 250 300 350 400
-1000 TSS +1000
d
Host (12,229)
H3K4me3
Pol II
Pol II
Fragment length (bp)
-100 TSS +100
e
HCMV (1,461)
H3K4me3
Pol II
Pol II
Fragment length (bb)

Supplementary Fig. 3. Visualizing transcription complexes and chromatin from DFF-Seq and Exp3 replicate data

a Length distribution of fragments +/- 1000 bp of each of 12,229 truQuant promoters for the Pol II and H3K4me3 DFF-ChIP datasets (Exp3). **b** Fragment count of selected ranges of fragments from the Pol II DFF-ChIP (Exp3) positioned across the 2000 bp region centered on the MaxTSS of 12,229 HFF truQuant promoters. The three curves were normalized by making the area under the curves equal. **c** fragMap representation of DFF-Seq data shown using two different black values for the 12,201 HeLa truQuant promoters showing fragments from 18-400 bp that are positioned +/-1000 bp around the MaxTSS. **d** FragMaps of H3K4me3 (Exp3) and Pol II (Exp3) for the 12,229 HFF truQuant promoters showing 18-400 bp fragments that are +/-1000 bp around the MaxTSS. A zoomed fragMap showing 18-120 bp fragments positioned +/-100 bp around the MaxTSS is also shown (right). **e** FragMaps of H3K4me3 (Exp3) and Pol II (Exp3) for the 1,461 TB40/E TSRs showing 18-400 bp fragments positioned +/-1000 bp around the MaxTSS. A zoomed fragMap showing 18-120 bp fragments positioned +/-100 bp around the TSS is also shown (right).

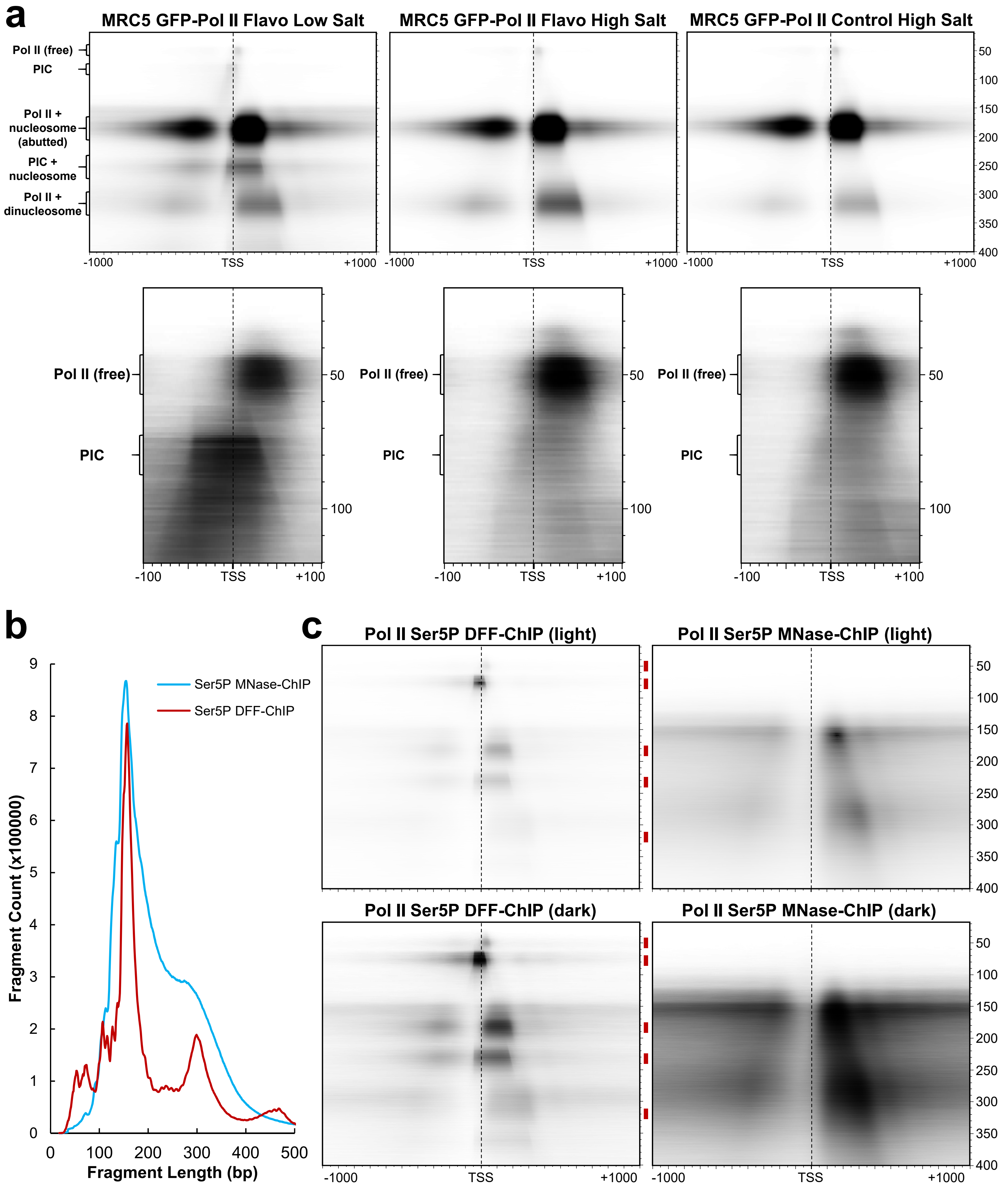
a
MRC5 GFP-Pol II Flavo Low Salt
MRC5 GFP-Pol II Flavo High Salt
MRC5 GFP-Pol II Control High Salt
Pol II (free)
PIC
Pol II + nucleosome (abutted)
PIC + nucleosome
Pol II + dinucleosome
-1000
TSS
+1000
50
100
150
200
250
300
350
400
Pol II (free)
PIC
-100
TSS
+100
b
Ser5P MNase-ChIP
Ser5P DFF-ChIP
Fragment Count (x100000)
Fragment Length (bp)
0
100
200
300
400
500
c
Pol II Ser5P DFF-ChIP (light)
Pol II Ser5P MNase-ChIP (light)
Pol II Ser5P DFF-ChIP (dark)
Pol II Ser5P MNase-ChIP (dark)

Supplementary Fig. 4. Salt sensitivity of TBP PICs on the host genome and inability of MNase-ChIP to detect transcription complexes.

a FragMaps of MRC5 GFP-Pol II datasets from Exp1 (flavo, low salt IP), Exp2 (flavo, high salt IP), and Exp2 (control high salt IP), showing 18-400 bp fragments positioned +/-1000 bp around the MaxTSS and zoomed in fragMaps showing 18-120 bp fragments positioned +/- 100 bp around the MaxTSS from 12,201 HeLa truQuant promoters. Fragment lengths of specified features are demarcated by brackets. For the wider fragMaps, the black value was reduced 3 fold to emphasize smaller fragments. For the zoomed in fragMaps, max black levels were set based on the max value in the shown region as standard for fragMaps. **b** Fragment distributions of the entire Exp4 Ser5P DFF-ChIP (red) or Ser5P MNase-ChIP libraries (blue)[52]. **c** FragMaps were created for Ser5P DFF-ChIP and Ser5P MNase-ChIP datasets and displayed with normal maximum black values (top) as well as with lower maximum black values to better visualize weaker features (bottom). Fragment sizes for transcription complexes (labeled in (**a**) that were missing in the MNase-ChIP fragMap as compared to the DFF-ChIP fragMap are marked by red boxes.

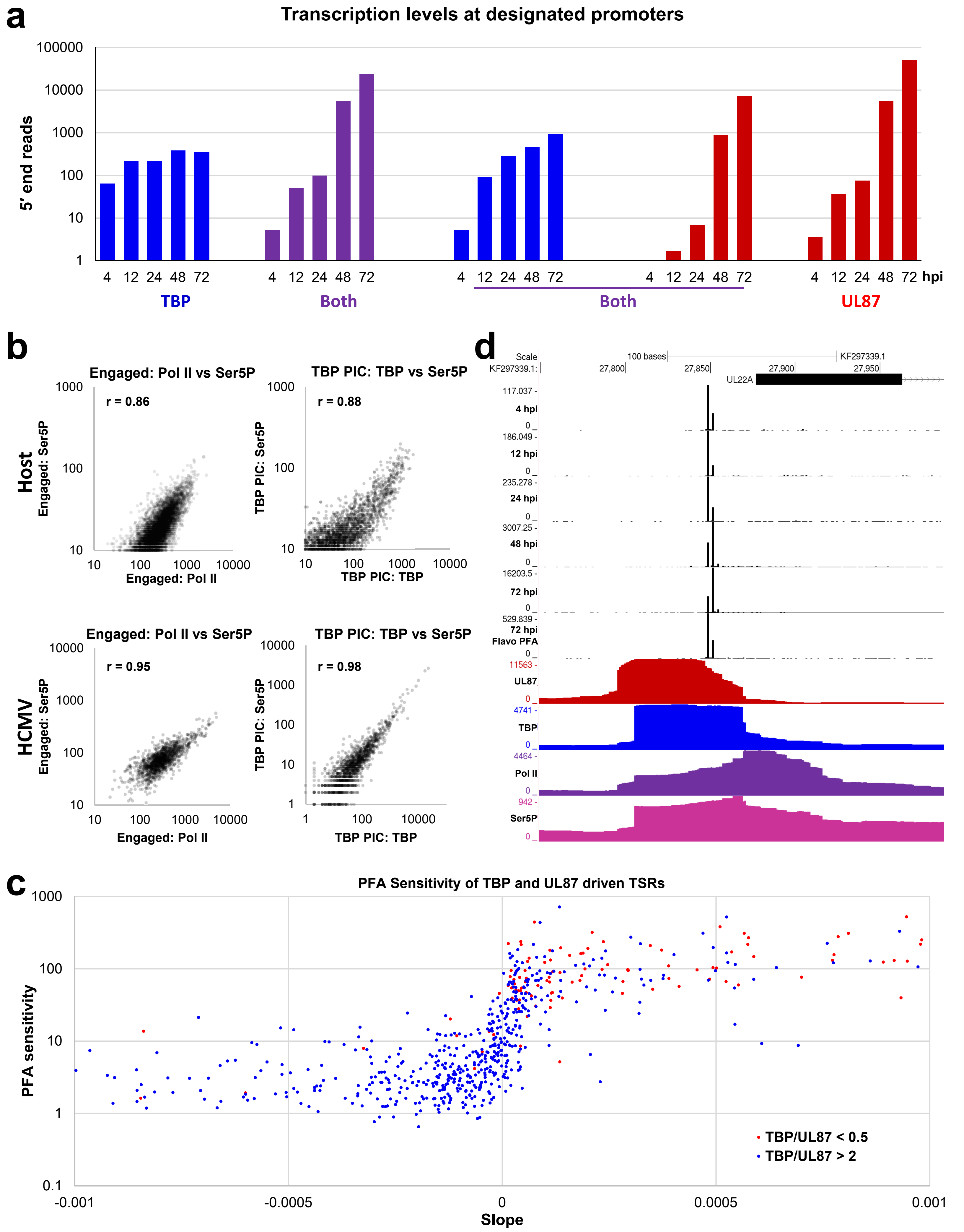
a
Transcription levels at designated promoters
5′ end reads
100000
10000
1000
100
10
1
4 12 24 48 72 hpi
TBP
Both
Both
UL87
b
Host
Engaged: Pol II vs Ser5P
r = 0.86
Engaged: Ser5P
Engaged: Pol II
TBP PIC: TBP vs Ser5P
r = 0.88
TBP PIC: Ser5P
TBP PIC: TBP
HCMV
Engaged: Pol II vs Ser5P
r = 0.95
Engaged: Ser5P
Engaged: Pol II
TBP PIC: TBP vs Ser5P
r = 0.98
TBP PIC: Ser5P
TBP PIC: TBP
d
Scale
100 bases
KF297339.1
KF297339.1:
27,800
27,850
27,900
27,950
UL22A
117.037 -
4 hpi
0
186.049 -
12 hpi
0
235.278 -
24 hpi
0
3007.25 -
48 hpi
0
16203.5 -
72 hpi
0
529.839 -
72 hpi
Flavo PFA
0
11563 -
UL87
0
4741 -
TBP
0
4464 -
Pol II
0
942 -
Ser5P
0
c
PFA Sensitivity of TBP and UL87 driven TSRs
PFA sensitivity
1000
100
10
1
0.1
-0.001
-0.0005
0
0.0005
0.001
Slope
TBP/UL87 < 0.5
TBP/UL87 > 2

Supplementary Fig. 5. TBP PICs are functionally different from UL87 PICs even though both may appear at late promoters

a Quantification of 5′ end reads (TSSs) in an 11 bp window around the five MaxTSSs specifically indicated in Figure 5A. **b** Correlations of the amount of engaged Pol II and TBP PIC features counted utilizing the Ser5P dataset in comparison to the Pol II and TBP dataset, respectively, on the host and HCMV genomes (Exp3). Pearson correlations (r) are provided. **c** TSRs with a TBP/UL87 ratio greater than 2 or less than 0.5 from Fig. 5B were plotted using their PFA sensitivity against the value of their slope calculated from the read counts from the 5 PRO-Seq time points. TSRs with a TBP/UL87 ratio greater than 2 were colored blue and TSRs with a ratio less than 0.5 were colored red. **d** Genome browser tracks depicting shifting 5′ ends for the UL22A promoter from PRO-Seq HCMV infection time course data and DFF-ChIP tracks showing UL87, TBP, Pol II, and Ser5P datasets (Exp4).

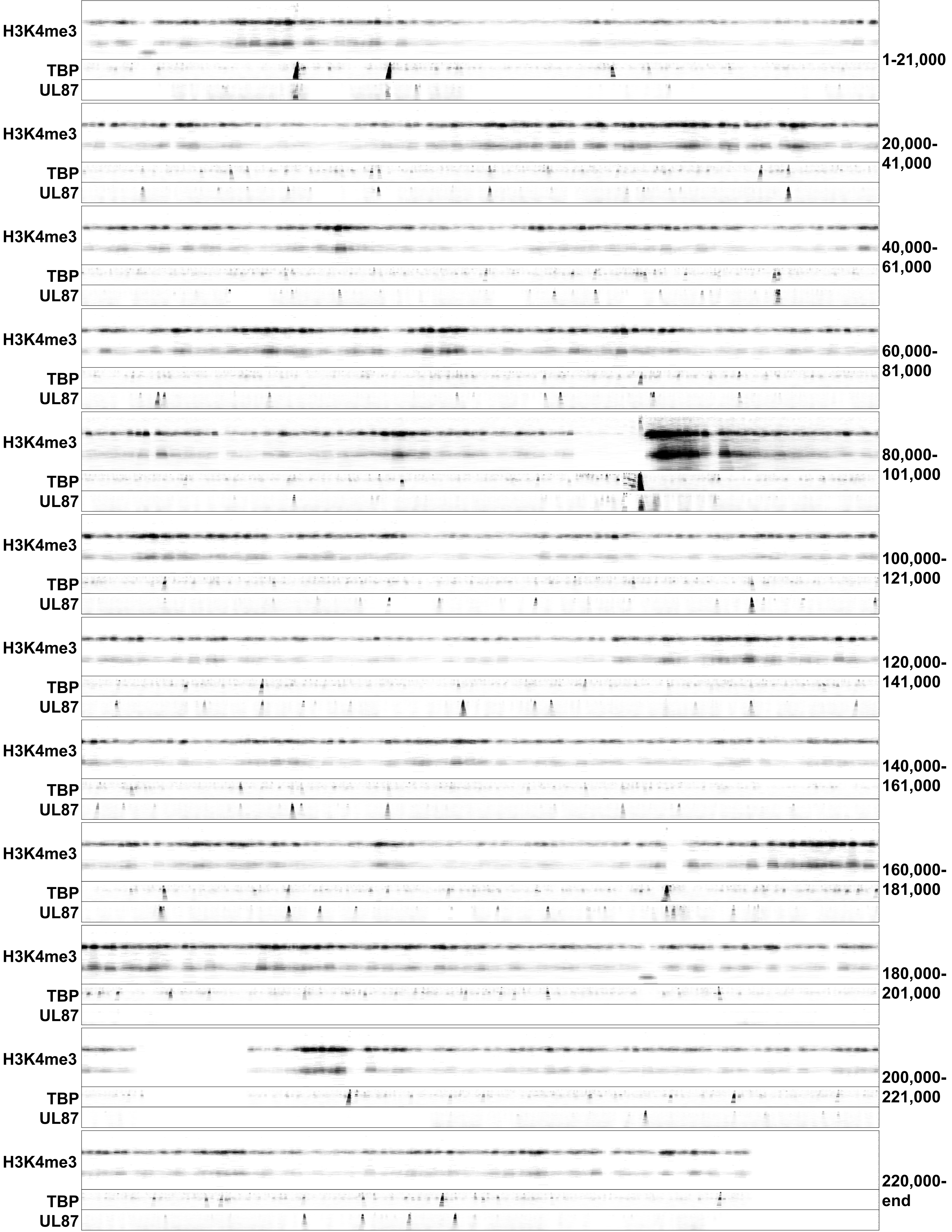

H3K4me3
TBP
UL87
1-21,000
20,000-41,000
40,000-61,000
60,000-81,000
80,000-101,000
100,000-121,000
120,000-141,000
140,000-161,000
160,000-181,000
180,000-201,000
200,000-221,000
220,000-end

Supplementary Fig. 6. Genomic fragMaps spanning the entire HCMV genome for H3K4me3, TBP, and UL87 fragments

Genomic fragMaps depicting overlapping 21,000 bp windows of the entire HCMV genome. H3K4me3 depict fragments between 18-400 bp whereas TBP and UL87 fragMaps depict fragments between 18-150 bp. Note that there is a 1000 bp overlap between each region shown to allow better visualization of features at the beginning of each region.

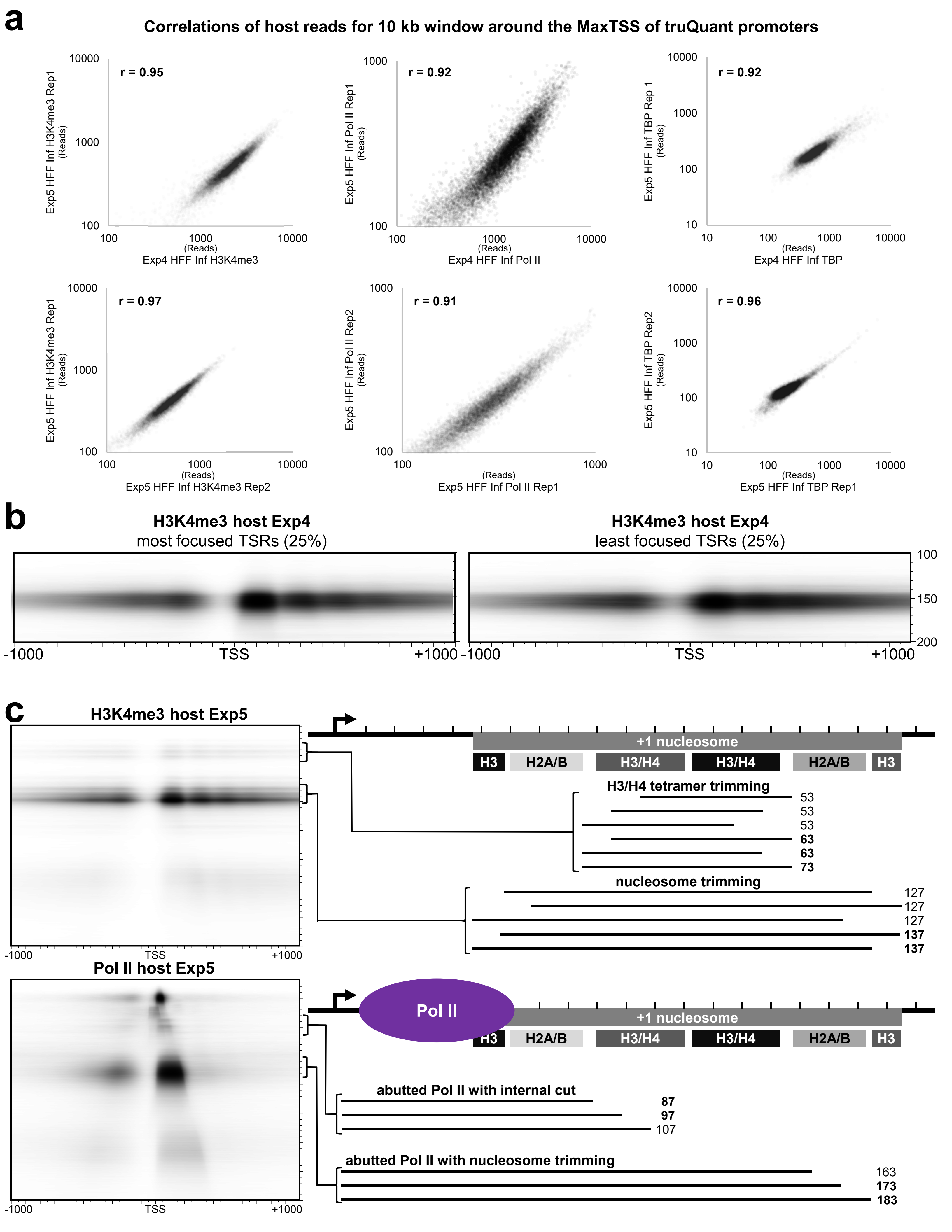

a
Correlations of host reads for 10 kb window around the MaxTSS of truQuant promoters
r = 0.95
r = 0.92
r = 0.92
r = 0.97
r = 0.91
r = 0.96
Exp5 HFF Inf H3K4me3 Rep1 (Reads)
Exp4 HFF Inf H3K4me3 (Reads)
Exp5 HFF Inf Pol II Rep1 (Reads)
Exp4 HFF Inf Pol II (Reads)
Exp5 HFF Inf TBP Rep 1 (Reads)
Exp4 HFF Inf TBP (Reads)
Exp5 HFF Inf H3K4me3 Rep2 (Reads)
Exp5 HFF Inf Pol II Rep2 (Reads)
Exp5 HFF Inf Pol II Rep1 (Reads)
Exp5 HFF Inf TBP Rep2 (Reads)
Exp5 HFF Inf TBP Rep1 (Reads)
b
H3K4me3 host Exp4
most focused TSRs (25%)
H3K4me3 host Exp4
least focused TSRs (25%)
-1000
TSS
+1000
100
150
200
c
H3K4me3 host Exp5
+1 nucleosome
H3
H2A/B
H3/H4
H3/H4
H2A/B
H3
H3/H4 tetramer trimming
53
53
53
63
63
73
nucleosome trimming
127
127
127
137
137
Pol II host Exp5
Pol II
abutted Pol II with internal cut
87
97
107
abutted Pol II with nucleosome trimming
163
173
183

Supplementary Fig. 7. Characterization of fragments resulting from excess DFF digestion
a Correlation plots comparing number of reads in 10,000 bp windows centered on the MaxTSS of 12,229 HFF truQuant promoters from the indicated Exp4 and Exp5 datasets. Pearson correlations (r) are provided. **b** H3K4me3 (Exp4) fragMaps from the top 25% and bottom 25% of the 12,229 truQuant promoters sorted by TSS focus (standard deviation of TSSs in TSRs). **c** fragMaps of H3K4me3 (Exp5) and Pol II (Exp5) showing 18-400 bp fragments positioned +/- 1000 bp around the MaxTSS containing from 12,229 HFF truQuant promoters with a schematic depicting the most likely nucleosome protections and DFF cleavage sites. Exp5 datasets are each a combination of two replicas (n = 2).
