# Supplementary material for: Differences in RNA polymerase II complexes and their interactions with surrounding chromatin on human and cytomegalovirus genomes
Source: Nat Commun. 2022 Apr 14;13:2006. doi: 10.1038/s41467-022-29739-x (PMC9010409; doi:10.1038/s41467-022-29739-x)
Supplement: Supplementary file 2 — Editorial Assessment Report [file 41467_2022_29739_MOESM2_ESM.pdf]

Contents of this report

1. **Manuscript details**: overview of your manuscript and the editorial team.
2. **Review synthesis**: summary of the reviewer reports provided by the editors.
3. **Editorial recommendation**: personalized evaluation and recommendation from all 3 journals.
4. **Annotated reviewer comments**: the referee reports with comments from the editors.
5. **Open research evaluation**: advice for adhering to best reproducibility practices.

About the editorial process

Because you selected the **Nature Portfolio Guided Open Access** option, your manuscript was assessed for suitability in three of our titles publishing high-quality work across the spectrum of genetics research: ***Nature Genetics***, ***Nature Communications***, and ***Communications Biology***. More information about Guided Open Access can be found here.

Collaborative editorial assessment

Your editorial team discussed the manuscript to determine its suitability for the Nature Portfolio Guided OA pilot. Our assessment of your manuscript takes into account several factors, including whether the work meets the **technical standard** of the Nature Portfolio and whether the findings are of **immediate significance** to the readership of at least one of the participating journals in the Nature Portfolio Guided Open Access genetics cluster.

Peer review

Experts were asked to evaluate the following aspects of your manuscript:

- **Novelty** in comparison to prior publications;
- **Likely audience** of researchers in terms of broad fields of study and size;
- **Potential impact** of the study on the immediate or wider research field;
- **Evidence** for the claims and whether additional experiments or analyses could feasibly strengthen the evidence;
- **Methodological detail** and whether the manuscript is reproducible as written;
- Appropriateness of the **literature review**.

Editorial evaluation of reviews

Your editorial team discussed the potential suitability of your manuscript for each of the participating journals. They then discussed the revisions necessary in order for the work to be published, keeping each journal's specific editorial criteria in mind.

Journals in the Nature portfolio will support authors wishing to transfer their reviews and (where reviewers agree) the reviewers' identities to journals outside of Springer Nature.
If you have any questions about review portability, please contact our editorial office at guidedoa@nature.com.

Manuscript details

Tracking number	Submission date	Decision date	Peer review type
GUIDEDOA-21-00267	Sep 16, 2021	Nov 23, 2021	Single-blind

Manuscript title

Differences in RNA polymerase II complexes and their interactions with surrounding chromatin on human and cytomegalovirus genomes

Preprint: There is a preprint of this manuscript available at Research Square.

Author details

David Price
Affiliation: University of Iowa

Editorial assessment team

**Primary editor**	**Tiago Faial**<br>Home journal: ***Nature Genetics***<br>ORCID: **0000-0003-0864-1200**<br>Email: **tiago.faial@us.nature.com**
**Other editors consulted**	**Poonam Bheda**<br>Home journal: ***Nature Communications***<br>ORCID: **0000-0001-6587-9456**<br><br>**George Inglis**<br>Home journal: ***Communications Biology***<br>ORCID: **0000-0002-9069-5242**
**About your primary editor**	Tiago Faial obtained his Ph.D. from the Stem Cell and Developmental Biology program at the University of Cambridge under the supervision of Jim Smith and Roger Pedersen, where he studied gene regulatory networks and signaling cascades that underpin mesoderm differentiation. For his postdoctoral work, Tiago joined Joanna Wysocka's laboratory at Stanford University where he studied the dynamics of epigenetic landscapes in pluripotency. He joined the *Nature Genetics* editorial team in 2015 and is based in San Francisco.

Editorial assessment and review synthesis

Editor's summary and assessment	SUMMARY<br>The authors perform chromatin immunoprecipitation (ChIP) for H3K4me3 and RNA Pol II after digestion with human DNA fragmentation factor (DFF) (DFF-ChIP; a new technique somewhat related to their run-on transcriptional profiling method [DFF-Seq] from ref. 6) in HeLa cells, MRC5 cells, and primary human foreskin fibroblasts infected with HCMV (human cytomegalovirus). Their analysis shows similarities and differences in RNA Pol II preinitiation complexes (PICs) driven by TBP on the host genome in comparison with PICs driven by TBP or by the viral-specific late initiation factor UL87 on the viral genome. Differences include the phosphorylation state of Pol II, the region of the promoter covered, and the lack of TFIIH.<br>They find that nucleosomes on the HCMV genome are irregularly spaced. Also, HCMV promoters are not surrounded by H3K4me3-marked nucleosomes. Host PICs and paused Pol II complexes are frequently in contact with the first downstream (+1) nucleosome. Paused Pol II is involved in the initial invasion of the +1 nucleosome. In contrast, viral transcription complexes have very limited nucleosomal interactions, likely reflecting a relative lack of chromatinization of transcriptionally active regions of HCMV genomes.<br><br>ASSESSMENT<br>The editors jointly decided to send this manuscript out to peer review based on the technical quality of the work, but there were some concerns from *Nature Genetics* that the topic and system were somewhat specialized, and that the approach may be too descriptive. Additionally, while the manuscript presents some new biological findings, these do not appear to be paradigm-shifting, largely building upon existing literature.<br>All editors noted that the benchmarking and comparison with competing methods could be improved.

**Editorial synthesis of reviewer reports**	Reviewer #1 thinks that the work is technically well done but that, despite the value of the new technique, the overall advance provided by this work is not sufficiently groundbreaking, which aligns with *Nature Genetics*'s initial assessment. This reviewer's comments are relatively minor and warrant a straightforward revision.<br>Reviewer #2 is positive about the work and they have mainly asked for some technical clarifications. Like Reviewer #1, they think the manuscript is densely written.<br>Reviewer #3's review is extremely detailed and constructive. The reviewer believes that the technique is potentially important, if widely adopted, but that the manuscript needs to be polished further.

Editorial recommendation

Nature Genetics<br><br>Revision not invited	The technique is interesting but the manuscript is descriptive, lacking sophisticated perturbation experiments that might provide more substantial mechanistic insights. The level of conceptual novelty (biological and technical) is not sufficiently disruptive to warrant publication in *Nature Genetics*.
Nature Communications<br><br>Major revisions	For consideration at *Nature Communications*, please include additional support for the chromatin state of the HCMV genome (Reviewers 1 and 3) and further validation of DFF and potential biases (Reviewers 2 and 3). In addition, all three reviewers commented on the readability of the manuscript and clarity/transparency of the analyses, especially of replicates. We would suggest improving the presentation in order to increase general interest and impact.
Communications Biology<br><br>Major revisions	While *Communications Biology* would not require the inclusion of any additional sequencing experiments or data, a revised manuscript should textually address all discussion points raised by the reviewers, as well as include the computational analyses outlined by Reviewers 1 and 3. Please also carefully proofread the manuscript to improve the overall readability and clarity of the results.

Next steps

**Editorial recommendation 1:**	Our top recommendation is to revise and resubmit your manuscript to *Nature Communications.* We feel the additional experiments required are reasonable.
**Editorial recommendation 2:**	You may also choose to revise and resubmit your manuscript to *Communications Biology.* This option might be best if the requested experimental revisions are not possible/feasible at this time.
**Note**	As stated on the previous page *Nature Genetics* is not inviting a revision. Please keep in mind that the journal will not be able to consider any appeals of their decision through Guided Open Access.

Revision

To follow our recommendation, please upload the revised manuscript files using **the link provided in the decision letter**.

Revision checklist

- ☐ Cover letter, stating to which journal you are submitting
- ☐ Revised manuscript
- ☐ Point-by-point response to reviews
- ☐ Updated Reporting Summary and Editorial Policy Checklist
- ☐ Supplementary materials (if applicable)

Submission elsewhere

If you choose not to follow our recommendations, you can still take the reviewer reports with you.

Option 1: Transfer to another Nature Portfolio journal
Springer Nature provides authors with the ability to transfer a manuscript within the Nature Portfolio, without the author having to upload the manuscript data again. To use this service, **please follow the transfer link provided in the decision letter.** If no link was provided, please contact guidedOA@nature.com.

> *Note that any decision to opt in to In Review at the original journal is not sent to the receiving journal on transfer. You can opt in to In Review at receiving journals that support this service by choosing to modify your manuscript on transfer.*

Option 2: Portable Peer Review option for submission to a journal outside of Nature Portfolio
If you choose to submit your revised manuscript to a journal at another publisher, we can share the reviews with another journal outside of the Nature Portfolio if requested. You will need to request that the receiving journal office contacts us at guidedOA@nature.com. We have included editorial guidance below in the reviewer reports and open research evaluation to aid in revising the manuscript for publication elsewhere.

Annotated reviewer reports

The editors have included some additional comments on specific points raised by the reviewers below, to clarify requirements for publication in the recommended journal(s). However, please note that all points should be addressed in a revision, even if an editor has not specifically commented on them.

**Reviewer #1 information**	
**Expertise**	transcriptional regulation of viral genomes
**Editor's comments**	This reviewer is supportive of the method but finds the biological insight limited and therefore provides suggestions for improving this aspect. This reviewer also highlights other aspects of the presentation that need to be improved.

**Reviewer #1 comments**	
**Section**	**Annotated Reviewer Comments**
**Remarks to the Author: Overall significance**	This study uses a novel approach called DFF-seq to examine host preinitiation complexes (PICs) and their relationship to chromatin in the human and HCMV genomes. DFF-seq essentially substitutes human DNA fragmentation factor (DFF) for sonication in ChIP-seq. They find differences in the fine structure of host and viral PICs, and that their relationship to chromatin (as inferred by the distribution of H3K4me3) is different. Not surprisingly they also found that H3K4me3 was not specifically distributed on the HCMV genome, and that PICS contains TBP and UL87 are somewhat different.
**Remarks to the Author: Impact**	The technique (DFF-seq) is powerful and will be of considerable value to the community. The biological information gained in this study is important, but represents a somewhat incremental advance compared to what we know.
**Remarks to the Author: Strength of the claims**	With respect to several points, further studies could be conducted to solidify and validate conclusions. Specific comments are below.<br><br>1. This paper is very dense with discussion of minutiae. It is light on important experimental details of the experiments being described. Too often they refer to "datasets", or experiments (ie: Exp3). It should be clear in the text and figure legends what exactly the experiment is (cell type, infected vs not infected, time after infection). This presents a bit of confusion particularly in fig 5A where pro-seq data for 5 time points is presented aligned in the same panel to DFF-seq for 4 proteins at 1 time point. Was DFF-seq ever done on infected cells prior to viral DNA replication?<br>**For the sake of reproducibility, please ensure that sufficient experimental detail is provided in the Methods such that readers could feasibly repeat these experiments. Other points related to reproducibility have been**

	**outlined in the Open Research Evaluation at the end of the Editorial Assessment Report.**<br><br>2. There is considerable attention to the nature of the H3K4me3 on the viral genome. It was concluded that its distribution and hence the location of nucleosomes on the viral genome was not restricted to active or inactive genes but distributed throughout the genome and possibly absent on active promoters. This is an issue in the field. This study needs to better explore the nature of that signal and the overall structure of the productive HCMV genome. Several questions:<br>a. It would be interesting to compare the fragment count vs fragment length distributions for an infected cell data set for the virus and cell in the same graph. Are di and tr nucleosome sized fragments less abundant on the viral genome?<br>b. Other histone antibodies should be used to be more comprehensive/representative of “nucleosomes/chromatin”, particularly for the viral genome.<br>c. ATAC-seq reveals mono, di, tri nucleosome fragment sizes on the cell genome. It would be useful for the authors to investigate this on the HCMV genome to validate the DFF seq size distribution data for the viral genome.<br>d. Why do TBP and polII show 150, 300, and 450 sized fragments in fig 7A?<br>e. Importantly, what is the nature of the relationships between PICs and “chromatin” on the viral genome prior to DNA replication? They should do DFF-seq at 4hpi.<br>**Points 2a-e would be necessary to address for consideration at *Nature Communications*. However, only points 2a, 2c, and 2d would have to be addressed for consideration at *Communications Biology* (2b and 2e could be mentioned as limitations throughout the text).**<br><br>3. The viral genome probably has many other viral proteins that contribute to its “chromatin”. These may or may not bind to the cell genome. One in particular, IE2, may contribute to the difference between viral and cellular PICs. DFF-seq should be done on IE2 and its relationship to PICs and the histone data. IE2 is probably a major driver of PIC formation on the viral genome.<br>**Although this point would not be necessary for consideration at Nature *Communications* or *Communications Biology*, it may help to further support additional analyses of HCMV chromatin state as required in Point 2.**<br><br>4. The DFF samples are not crosslinked as in ChIP. Why is that? One can do ATAC seq on crosslinked samples.
**Remarks to the Author: Reproducibility**	The appropriate statistics and data analysis was applied.

Reviewer #2 information	
**Expertise**	transcriptional regulation of mammalian genomes
**Editor's comments**	This reviewer points out a few further validations of the method that would improve the reliability of the results. This reviewer also highlights other aspects of the presentation that need to be improved.

Reviewer #2 comments	
**Section**	**Annotated Reviewer Comments**
**Remarks to the Author: Overall significance**	The manuscript by Spector and colleagues studies the relationship between nucleosomes, the PIC, and the Pol II elongation complex in human cytomegalovirus (HCMV) and human host genomes. The authors introduce DFF-ChIP, a new technique in which they digest chromatin with DNA fragment factor (DFF) and perform immunoprecipitation for Pol II, TBP, or modified histones. The authors separate fragments based on the size of DNA protected from digestion and their position relative to transcription start site. The authors find evidence for a variety of different complexes on chromatin, including the PIC, elongation complex in which Pol II is either alone, or is complexed partially in transit across the +1 nucleosome. Last, the authors show that these different PIC and elongation complexes differ significantly in the human and HCMV genome. Overall, the manuscript is densely written and somewhat challenging to read. However, the content is interesting and the analysis appears mostly to be solid (pending some additional validation of DFF and a few other issues). In particular, I am enthusiastic that DFF-ChIP appears to provide such solid information about the PIC, providing the opportunity to study its relationship with both paused Pol II and other elongation complexes.<br><br>This work should be published provided that the authors address several major points listed below.<br><br>* Please add Y-axis labels to Fig. 2 A and B and a color scale to all of the fragMap heatmaps. Finally, can the authors please clarify that the Y scale on H3K4me3 fragMaps is the same as the scale provided on Pol II? These are really required to adequately evaluate several of the author's interpretations.<br><br>* The authors should clearly discuss the rationale for using DFF instead of alternative enzymes which are more widely used for similar purposes (e.g., MNase). I'm sure DFF has some advantages; these would be useful for readers to understand.<br><br>I'm sure DFF has some disadvantages as well. For instance, I don't believe DFF

is an exonuclease; so can we trust that the edges of fragments represent the boundary of protection? The fragMaps look promising, but this is not discussed or validated in the paper. There should be more validation presented about how DFF protection relates to footprint size.

This point would be necessary to address for consideration at *Nature Communications* and *Communications Biology*.

Likewise, how much space is required for DFF to cut DNA? The argument that paused Pol II was "abutting" a nucleosome depends a great deal on this. Could Pol II be upstream, with no direct contact with the nucleosome, but there just isn't quite enough space for DFF to cut?

* The caption to Fig. S1 lists a comparison to MNase-seq. I think it would be helpful to show this, but I don't think this element of the figure was included in the Fig. Fig. S1 in my PDF?! And I did not see this mentioned elsewhere in the text.

* Fig. 4B (the Ser5P fragMap) is interesting. The authors argue that Fig. 4B is the "correct" distribution due to epitope masking of the antibody they use for total Pol II. But this image would suggest that the vast majority of Pol II is in the PIC, rather than in the free pause. This result would be quite surprising and would seem to contradict a number of papers, not all of which use antibodies that could be fooled by epitope masking. However, the authors don't show the full Ser5P fragMap. One possible way to reconcile the fragMaps the authors show with the previous literature is that Ser5P is strong in the nucleosome "abutted" pause. Can the authors please show this entire fragMap in the supplement? If this does explain the difference, it would seem that the 50 bp band does not reflect paused Pol II (which should have high levels of Ser5P) - and so if this is the case, what is the 50 bp Pol II?

Please clarify this point for further consideration at *Nature Communications* and *Communications Biology.*

* The authors cite Fig. 3A as the figure shows TBP DFF-ChIP (on pp. 6). This should be 4A.

Minor

* Overall, I like the FragMaps and find them to be a useful visualization. However, the black/ gray/ white color scale looks so much like Western blot in some of the panels that I worry some readers will find it confusing. This comment is completely up to the authors whether they would like to address or not.

Editors would also leave this point up to the authors' discretion.

* In some cases, figure titles should be stated more clearly. For instance, titles of panel A and B in Fig. 7 state "Fragment distribution Exp4" and "Fragment

	distribution Exp5”. I would find it easier to read the paper if the titles more clearly indicated the difference between Exp4 and Exp5 was the amount of digestion.

Reviewer #3 information

**Expertise**	RNA polymerase II function
**Editor’s comments**	This reviewer is supportive of the method but finds the impact moderate, mainly due to presentation, and offers suggestions for improving this aspect.

Reviewer #3 comments

Section	Annotated Reviewer Comments
**Remarks to the Author: Overall significance**	Spector and colleagues present a manuscript entitled “Differences in RNA polymerase II complexes and their interactions with surrounding chromatin on human and cytomegalovirus genomes” where they employ a novel technique for chromatin fragmentation using the “DNA Fragmentation Factor” nuclease. DFF has properties that make it attractive alternative to micrococcal nuclease in that it has much decreased purported ability to cut within nucleosomal DNA and this restraint on its cleavage enables it to useful probe large complexes associated with DNA. This is an incredibly exciting technique that will have huge value if generally employable. Here they employ DFF to understand the distribution of RNA Polymerase II complexes at transcription start sites and how these complexes might be associated with nucleosomes. As a comparative model, they exploit HCMV infection of human cells and examine the viral genome for transcription complexes of different types. The strengths of the manuscript are the new technology that can be used to example distinct complexes at different genes and the wealth of biology it might be applied to in the future. The drawbacks are that the manuscript feels like two distinct themes artificially joined together in a presentation that does not make the work accessible to readers generally interested in gene expression. The HCMV work seems grafted on with suboptimal treatment or description of the impact of the experiments to what is known and somewhat by the limitations in experimental choices for analysis. The manuscript is a bit rough and organization and clarity could be improved with some dedicated editing.
**Remarks to the Author: Impact**	If presentation is improved this work can have significant impact. Currently the impact is moderate.
**Remarks to the**	Major issues:

<table>
<tr>
<td>Author: Strength of the claims</td>
<td>
1. The manuscript is not accessible to the general reader. The framework and overview of the work is not forcefully presented. The experiments presented seem somewhat of a report of things that were tried and what results were that is more casual than might be expected for the first description of a novel technique coupled with clear demonstrations of key aspects. As an example, it is totally unclear what experiments in the paper are actually reproducible by standard definition of reproducibility, that is, biological replicate experiments. There is no doubt in my mind that this approach is highly likely to be highly reproducible, but it is not necessarily appropriate to take samples representing different treatments and showing they correlate. This would not be a demonstration of reproducibility, though high correlate between two different samples might be predictive of expected reproducibility. It is generally expected that experiments are done in duplicate on biological replicates. This is really the minimum. It appears that conditions change between some experiments and there are some conditions that are not discussed so it is not clear if any experiments are replicated or not.
Transparency regarding replicates is necessary for further consideration at Nature Communications and Communications Biology. Please also refer to the Open Research Evaluation at the end of the Editorial Assessment Report.

2. There are many examples where phrases are used without clear definition at first use, while defined later. This could potentially frustrate readers and minimize the impact of the work.

3. There seems that either the biology of the HCMV system could be made more apparent or it could be discussed simply as a paradigm to understand how different initiation mechanisms might be appreciated using DFF-ChIP, and then presented in this way throughout the manuscript. Currently it feels in between because the presentation is not cohesive.

4. Perhaps authors are doing this analysis elsewhere, but while the combining all promoters together for most of the FragMaps is interesting, it obscures how the different populations of fragments arise and whether they are arising from distinct promoters or promoter classes. This makes it less clear how to actually interpret the combined FragMaps as they would relate to individual promoters. This is not really addressed in anyway. One possibility is to more clearly justify or lay out the case for the value of the FragMaps that use all the data from 12K TSSs at one time. Whatever the authors choices are, they should be made as transparent as possible for the reader and as consistent as possible. There is essentially a single promoter level analysis that is opaque (Figure 3E) in depth of data at individual promoter level.
Please clarify this point for further consideration at Nature Communications and Communications Biology.
</td>
</tr>
</table>

	Other suggestions:<br><br>5. Introduction. Promoters are made to appear relatively homogeneous in structure, for example, positioning of nucleosome downstream of TSS and paused Pol II. Is this the case? The technique developed here has power to quantitatively analyze promoter classes/subclasses.<br><br>6. p2. “Occupancy by the +1 nucleosome is near 100% while Pol II occupancy is generally much less than 10%, resulting in a potentially misleading correlation between two disparate signals.”<br><br>What are the data that indicate +1 is 100% occupancy and for what fraction of promoters? These estimates should have citations.<br><br>7. p2. “Recently there has been”<br><br>“has” -> “have”<br><br>Results<br><br>8. The treatment of the DFF sequence specificity does not seem adequate, as the sequence preference seems more than slight and the analysis for percentage that have putative consensus isn’t explained or shown. Could this be given as a sequence logo and also potentially a color map (by base) indicating some number of cut sites?<br>**Please clarify this point for further consideration at *Nature Communications* and *Communications Biology.***<br><br>9. p3 “to explore its use as a front end” -> jargon<br><br>10. p3 “Nuclei from non-crosslinked HeLa or MRC5 cells expressing a GFP-tagged Pol II were digested with DFF for 1 hour to generate primarily mononucleosomes.”<br><br>Are the samples different between the H3K4Me chip (HeLa flavo, MRC5, MRC5 flavo) vs GFP-Pol II (see labels, i.e. what is HS?). It is entirely unclear which if any of these samples should be considered replicates or not- they are introduced as indicating reproducibility when it appears they are different samples that show signal that is highly correlative, as in they are similar.<br>**Transparency regarding replicates is necessary for further consideration at *Nature Communications* and *Communications Biology.***<br><br>11. p3 “Unlike the host genome where H3K4me3 is found only around promoter regions, the entirety of the HCMV genome is covered with H3K4me3, at levels ranging from relatively low to more enriched irrespective of Pol II occupancy.”

The choice of H3K4me3 for host promoters makes sense, but once it was determined that H3K4me3 was distributed differently on the HCMV genome, it becomes much more important to treat these data very carefully in terms of inference. It does not seem appropriate that the lack of this modified nucleosome should be used to infer what the chromatinized state of the HCMV genome is. I recognize that the widespread transcription observed is consistent with lower nucleosome occupancies, but in the absence of having idea of what fractional occupancies are of total nucleosomes, authors should be much more conservative in discussion of what H3K4me3 distribution on HCMV means. It primarily can be interpreted that it is not used in the same fashion and the low levels (relative to genome copy number) suggest that it does not have major function at all.

As Reviewer 1 made suggestions to improve this aspect of the manuscript, this point should be further investigated/supported for consideration at *Nature Communications* and *Communications Biology*.

12. At the very first use of terms “free” and “abutted” please make it as clear as possible in a systematic way that fragments of specific size are being used to infer Pol II complex types and what they are. It would be more conservative to talk about fragment sizes and attribution consistently through the manuscript and also to label these putative classes on FragMaps or to have a figure indicating what the attributions are.

13. p4 “The total amount of reads in each of these ranges was normalized to emphasize the protected footprints of the less abundant ~75 bp fragments.”

I am not certain what this means. I think what is meant is the read totals in these specific ranges were used for normalization instead of total reads for all fragment sizes.

14. p4 “Since metaplots limit how many fragment lengths can be shown together in a meaningful and accurate way, we created a method that more holistically depicts the distribution of all fragments around TSSs.”

I would recommend describing where the TSSs came from after this sentence, not several sentences later. I would also consider this comment below for rephrasing and repositioning (it comes later in the manuscript in the middle of a paragraph). First, it is sort of hard to understand. Second, it seems like it should be mentioned clearly and in section describing how FragMaps work.

“The TSRs utilized to generate HCMV fragMaps are 200 bp so individual TSRs may be represented multiple times in the 2000 bp window. This causes a light background of particularly sized fragments across the entire visualized region.”

15. p5 “Examination of the fragment count of +1 nucleosome sized fragments

from the H3K4me3 dataset shows that the host promoters with the absolute highest percentage of free Pol II have little +1 nucleosome, but most promoters have a similar amount of +1 nucleosome"

This is one of the few comments that examines individual promoters, but it does not quantify and settles for "most". Also if the graphs on the right are rank ordered the same as on the left, this should be made absolutely clear in the figure. Figures and legends should be made as clear as possible.

16. p6 "There is a difference in the relative amounts of PIC and free paused Pol II detected with the F12 antibody and the Ser5P antibody."

This is an example of how the manuscript is not effective at introducing concepts or structuring text for reader accessibility. The mention of the F12 antibody comes out of nowhere- it is not clear which samples are F12, there is no label on any figure that I can determine indicates F12 use.

17. p6. "As with the free-standing PIC, the PIC/+1 nucleosome feature on the host genome
was salt sensitive (Supplementary Fig. 4C)."

What is the basis for this determination? Any quantitation? These features appear to be less salt sensitive than other features described as salt sensitive. At first use of the term feature the definition of feature (a cluster of fragments within a certain size range and promoter position) and the attributions for each feature should be given ("x feature is attributed to paused Pol II that is not associated with an adjacent nucleosome "free Pol II", y feature is attributed to…).

18. p7 "UL87 evidently has a stricter requirement for TATT containing sequences with 128/146 matches to its HCMV Logo, while TBP had a lower percentage of matches to its host (623/1230) and viral (88/146) Logos."

What is the definition of a match to logo? The most preferred base?

19. p7 "Representative regions of the HCMV genome containing early and late genes are depicted in a 1,400 bp region and an 800 bp region with corresponding HCMV genomic fragMaps below showing fragments from the UL87, TBP, Pol II, and Ser5P datasets (Fig. 5A)."

It is not clear what are in these FragMaps. Is this just Exp 4 datasets? Were their timepoints for DFF experiment or just for PRO-seq?

20. p7. "Quantification of 5′ end reads found in an 11 bp window around the MaxTSS of these five promoters shows that TBP-driven TSSs are more active early in comparison to UL87 PICs that are active late (Supplementary Fig. 5A).

Because some promoters are driven by both TBP and UL87 the distinction between early versus late gene transcription is more complex than a simple separation of TBP and UL87 driven promoters.”

This lacks clarity because it is said that some promoters have the same TSS for TBP and UL87 and that TSRs were used for the viral genome and MaxTSS was not previously used to indicate viral TSS. I assume what is being done here is that TSSs that appear dominated by TBP binding in [specific dataset] show activity by PRO-seq earlier and that TSSs dominated by UL87 in [specific dataset] show activity by PRO-seq later.

21. p7 “The fractional usage for each promoter at each time point was displayed in a heatmap after normalizing to the total number HCMV reads in each time point. 795 of the 1,461 promoters that each had a total of 100 reads across the time course were then sorted based on PFA sensitivity.”

As an example of how organization and rationale are opaque, PFA is first introduced here and is not defined until a number of sentences later.

22. p7 “The three sorts gave similar patterns of genes with early (red to green) and late (green to red) transcription kinetics.”

Are these three datasets sorted the SAME way or three datasets sorted three different ways? This is not clear to the reader and the figure labeling does not aid reader either.

23. p10 “Although it is likely that the large majority of HCMV promoters do not feature modified nucleosomes immediately downstream, it is not possible to prove that this is the case at every individual viral promoter. Post-translationally modified nucleosomes may be positioned on individual loci at some stages of the viral life cycle22,23. Overall, we conclude that during lytic infection the HCMV genome is transcribed in a predominantly nonchromatinized state.”

The strongest data here would be showing lack of Pol II complexes in the 150-200 range- but this is never shown. What is the justification that H3K4me should be used to make conclusions about all nucs on HCMV genome? It seems that when “modified” is used in this sentence this is shorthand for “H3K4me3” nucleosomes, but there seems to be little or no justification provided to use a single dataset for nucleosome species marked with H3K4me3 to make conclusions about the viral genome chromatin state.

> **As Reviewer 1 made suggestions to improve this aspect of the manuscript, this point should be further investigated/supported for consideration at *Nature Communications*. As stated above, however, additional sequencing experiments would not be necessary for consideration at *Communications Biology*, so long as limitations are**

	**appropriately discussed.**<br><br>24. p10 "In contrast, UL87 PIC footprints are only located upstream of the TSS, suggesting that UL87 PICs lack the subunits of TFIID that contact downstream DNA and crucially, at least the XPB subunit of TFIIH."<br><br>Why is the conclusion that subunits are lacking rather than subunit-DNA interactions? Arguments are reasonable about TFIIH but should be more conservative for TFIID.<br><br>25. Figure 1A. Correlation between samples treated differently reveals similarity in behavior with the assay not reproducibility. Sample labels are not explained.<br><br>26. Figure 1D. What are the units of the graphs? No graph should lack units.<br><br>27. Figure 3B. "Total fragment length counts were normalized" This is unclear. I assume what is meant is fragment length counts across fragment lengths were normalized to total fragment length counts"<br><br>28. Figure 3D. "fragMaps of H3K4me3 and Pol II for the 1,461 TSRs showing fragments from 18-400 bp that are +/-1000 bp relative to the MaxTSS."<br><br>"for the 1,461 TSRs" – make it clear i.e. "1,461 transcription start regions (TSRs) identified in the HCMV genome". Similarly when referring to "truQuant genes" consider "length distribution…shown for fragments +/- 1000 bp from the TSS of greatest usage for 12,229 genes identified…."<br><br>29. Figure 3E. Since most FragMaps are accumulated signal from many genes, the coverage of individual TSS regions, which is where the numbers used for 3E will come from are unclear. There should be supplemental figure of some sort illustrating reads mapped per region and for this figure make it clear what numbers are or if there is a cutoff for read coverage employed what is it.<br><br>"Utlizing the same sort" -> please be specific.<br><br>30. Figure 4C. Is normalization changing when fragment size is changing or are just different fragment sizes being displayed?
**Remarks to the Author: Reproducibility**	Reproducibility is addressed in comments above.

Open research evaluation

Data availability
**Data availability statement**
Please add a Data Availability statement. Please ensure that your Data Availability statement includes accession details for deposited data, mentions where Source data can be found, and states that all other data are available from the corresponding author (or other sources, as applicable) on reasonable request.<br><br>More information about our data availability policy can be found here: https://www.nature.com/nature-portfolio/editorial-policies/reporting-standards#availability-of-data<br><br>See here for more information about formatting your Data Availability Statement: http://www.springernature.com/gp/authors/research-data-policy/data-availability-statements/12330880
**Mandatory data deposition**
For all sequencing data, submission to a community-endorsed, public repository is mandatory for publication in a Nature Portfolio journal and is best practice for publication in any venue. Accession numbers must be provided in the paper. Examples of appropriate public repositories are listed below:<br><br>-GenBank (all DNA sequence data)<br>-Sequence Read Archive (high-throughput sequence data)<br>-Gene Expression Omnibus (Microarray or RNA sequencing data)<br><br>More information on mandatory data deposition policies at the Nature Portfolio can be found at http://www.nature.com/authors/policies/availability.html#data<br><br>Please visit https://www.springernature.com/gp/authors/research-data-policy/repositories/12327124 for a list of approved repositories for each mandatory data type.
Our journals strongly support public availability of data and custom code associated with the paper in a persistent repository where they can be freely and enduringly accessed or as a supplementary data file when no appropriate repository is available. If data and code can only be shared on request, please explain why in your data Availability Statement, and also in the correspondence with your editor. For more information, please refer to https://www.nature.com/nature-research/editorial-policies/reporting-standards#availability-of-data

Please ensure that datasets deposited in public repositories are now publicly accessible, and that accession codes or DOI are provided in the "Data Availability" section. As long as these datasets are not public, we cannot proceed with the acceptance of your paper. For data that have been obtained from publicly available sources, please provide a URL and the specific data product name in the data availability statement. Data with a DOI should be further cited in the methods reference section.

Reporting & reproducibility

We encourage you to share your step-by-step experimental protocols on a protocol sharing platform of their choice. The Nature Portfolio's Protocol Exchange is a free-to-use and open resource for protocols; protocols deposited in Protocol Exchange are citable and can be linked from the published article. More details can be found at www.nature.com/protocolexchange/about

Please state in the legends how many times each experiment was repeated independently with similar results. This is needed for all experiments, but is particularly important wherever results from representative experiments (such as micrographs) are shown. If space in the legends is limiting, this information can be included in a section titled "Statistics and Reproducibility" in the methods section.

Statistics and data presentation

To improve reproducibility of your analyses, please provide details regarding your treatment of outliers.

Wherever statistics have been derived (e.g. error bars, box plots, statistical significance) the legend needs to provide and define the n number (i.e. the sample size used to derive statistics) as a precise value (not a range), using the wording "n=X biologically independent samples/animals/cells/independent experiments/n= X cells examined over Y independent experiments" etc. as applicable.

Statistics such as error bars, significance and p values cannot be derived from n<3 and must be removed from all such cases.

We strongly discourage deriving statistics from technical replicates, unless there is a clear scientific justification for why providing this information is important. Conflating technical and biological variability, e.g., by pooling technically replicates samples across independent experiments is strongly discouraged. (For examples of expected description of statistics in figure legends, please see the following https://www.nature.com/articles/s41467-019-11636-5 or https://www.nature.com/articles/s41467-019-11510-4).

All error bars need to be defined in the legends (e.g. SD, SEM) together with a measure of centre (e.g. mean, median). For example, the legends should state something along the lines of "Data are presented as mean values +/- SEM" as appropriate. All box plots need to be defined in the legends in terms of minima, maxima, centre, bounds of box and whiskers and percentile.

The figure legends must indicate the statistical test used. Where appropriate, please indicate in the figure legends whether the statistical tests were one-sided or two-sided and whether adjustments were made for multiple comparisons. For null hypothesis testing, please indicate the test statistic (e.g. F, t, r) with confidence intervals, effect sizes, degrees of freedom and P values noted. Please provide the test

results (e.g. P values) as exact values whenever possible and with confidence intervals noted.
**Other notes**
We have included as an attachment to the decision letter a version of your Reporting Summary with a few notes. This is mainly for your information, but we hope it is helpful when preparing your revised manuscript. If you decide to resubmit the manuscript for further consideration, please be sure to include an updated Reporting Summary.
