# Supplementary material for: Differences in RNA polymerase II complexes and their interactions with surrounding chromatin on human and cytomegalovirus genomes
Source: Nat Commun. 2022 Apr 14;13:2006. doi: 10.1038/s41467-022-29739-x (PMC9010409; doi:10.1038/s41467-022-29739-x)
Supplement: Supplementary file 3 — List of supplementary information [file 41467_2022_29739_MOESM3_ESM.docx]

**Title:**

Supplementary Data 1 file

**Legend:**

This Excel file contains 5 types of information. **Library Statistics** provides information on the workup of both DFF-ChIP and PRO-Seq libraries that includes mapping statistics to appropriate genomes, final read counts, and in the case of PRO-Seq libraries the correction factors generated using spike-ins. **Feature Descriptions** provides criteria for the features discussed and quantified. The range of positions and fragment lengths useful for visualization on fragMaps is listed in the table "Feature criteria for viewing fragMaps." For quantification of features from particular datasets fragment centers were used which allows an improvement in resolution. A fragment must both be of the correct size and have a center that falls in the specified window. These features are found in the table "Feature criteria for quantifactions." **Quantification of features** is provided in multiple datasheets with names containing an experiment number, a genome, and the word “Features.” **Specific analyses** are provided in the following datasheets which contain analyses using data from the quantification of features or from the PRO-Seq data. **Blocklist for hg38:** hg38.GencodeV27.miRNA-scRNA-snRNA-snoRNA-rRNA-scaRNA-tRNA-rDNA.padded50bp.lsu_ssu
