# Supplementary material for: Differences in RNA polymerase II complexes and their interactions with surrounding chromatin on human and cytomegalovirus genomes
Source: Nat Commun. 2022 Apr 14;13:2006. doi: 10.1038/s41467-022-29739-x (PMC9010409; doi:10.1038/s41467-022-29739-x)
Supplement: Supplementary file 5 — Reporting Summary [file 41467_2022_29739_MOESM5_ESM.pdf]

nature research

Corresponding author(s): David H. Price
Last updated by author(s): 2/17/2022

Reporting Summary

Nature Research wishes to improve the reproducibility of the work that we publish. This form provides structure for consistency and transparency in reporting. For further information on Nature Research policies, see our Editorial Policies and the Editorial Policy Checklist.

Statistics

For all statistical analyses, confirm that the following items are present in the figure legend, table legend, main text, or Methods section.

n/a	Confirmed	
☒	☐	The exact sample size (n) for each experimental group/condition, given as a discrete number and unit of measurement
☐	☒	A statement on whether measurements were taken from distinct samples or whether the same sample was measured repeatedly
☒	☐	The statistical test(s) used AND whether they are one- or two-sided<br>*Only common tests should be described solely by name; describe more complex techniques in the Methods section.*
☒	☐	A description of all covariates tested
☒	☐	A description of any assumptions or corrections, such as tests of normality and adjustment for multiple comparisons
☒	☐	A full description of the statistical parameters including central tendency (e.g. means) or other basic estimates (e.g. regression coefficient) AND variation (e.g. standard deviation) or associated estimates of uncertainty (e.g. confidence intervals)
☒	☐	For null hypothesis testing, the test statistic (e.g. F, t, r) with confidence intervals, effect sizes, degrees of freedom and P value noted<br>*Give P values as exact values whenever suitable.*
☒	☐	For Bayesian analysis, information on the choice of priors and Markov chain Monte Carlo settings
☒	☐	For hierarchical and complex designs, identification of the appropriate level for tests and full reporting of outcomes
☐	☒	Estimates of effect sizes (e.g. Cohen's d, Pearson's r), indicating how they were calculated

Our web collection on statistics for biologists contains articles on many of the points above.

Software and code

Policy information about availability of computer code

Data collection	Library sequencing was performed on Illumina HiSeq 4000 or Novaseq 6000 using Illumina software bcl2fastq2 (version 2.2)
Data analysis	DNAfastqtoBigWig (https://github.com/P-TEFb/DNAfastqtoBigWig)<br>trim_galore 0.6 (https://github.com/FelixKrueger/TrimGalore/releases/tag/0.6.6)<br>ABD.py (https://github.com/P-TEFb/ABD)<br>fragMap.py (https://github.com/P-TEFb/fragMap)<br>truQuant (https://github.com/meierjl/truQuant)

For manuscripts utilizing custom algorithms or software that are central to the research but not yet described in published literature, software must be made available to editors and reviewers. We strongly encourage code deposition in a community repository (e.g. GitHub). See the Nature Research guidelines for submitting code & software for further information.

Data

Policy information about availability of data

All manuscripts must include a data availability statement. This statement should provide the following information, where applicable:
- Accession codes, unique identifiers, or web links for publicly available datasets
- A list of figures that have associated raw data
- A description of any restrictions on data availability

Raw and processed sequencing data generated for this manuscript can be obtained from GEO (GSE185763) without restriction. Previously published PRO-Cap datasets of uninfected HFFs and 96 hpi Towne infected HFFs (GSE113394) and HeLa NasCap data (GSE139237) are available from GEO without restriction.
Fig. 1 GSE185763

Fig. 2 GSE185763
Fig. 3 GSE185763, GSE139237, and GSE113394
Fig. 4 GSE185763, GSE139237, and GSE113394
Fig. 5 GSE185763 and GSE113394
Fig. 6 GSE185763, GSE139237, and GSE113394
Fig. 7 GSE185763 and GSE139237
Supplemental Figures GSE185763, GSE139237, and GSE113394

Field-specific reporting

Please select the one below that is the best fit for your research. If you are not sure, read the appropriate sections before making your selection.

☒ Life sciences ☐ Behavioural & social sciences ☐ Ecological, evolutionary & environmental sciences

For a reference copy of the document with all sections, see nature.com/documents/nr-reporting-summary-flat.pdf

Life sciences study design

All studies must disclose on these points even when the disclosure is negative.

Sample size	No statistical methods were used to predetermine sample size. PRO-Seq, PRO-Cap, and DFF-ChIP datasets started with samples of from ~5 -10 million cells to ensure robustness and to minimize cell to cell differences.
Data exclusions	For analysis of TSSs on the host genome we used a modified blocklist hg38.GencodeV27.miRNA-scRNA-snRNA-snoRNA-rRNA-scaRNA-tRNA-rDNA.padded50bp.lsu_ssu that is provided in the Supplementary Data file (hg38 blocklist) to exclude interference from Pol I and Pol III transciption and related repetitive regions because we were examining Pol II transcription.
Replication	The point of our study was to demonstrate that the new method we developed (DFF-ChIP) could identify transcription complexes and their interaction with nearby chromatin. Because it was not primarily to quantify subtle changes, exact replication of all experiments was not required. Experimental differences between some datasets strengthen our results which highlight the similarity between samples regardless of the perturbations which include the use of three different cell types and various treatment conditions. In one section we do make comments about differences (loss of PICs at high salt) and that figure shows duplicated datasets. A Supplemental Data file contains a color coded guide as to what the similarities and differences were between samples which makes it easy determine which datasets provide evidence of reproducibility.
Randomization	Samples were applied to the sequencing flow cell to randomize localization
Blinding	The investigators were not blinded during data acquisition or analysis as this is not required for the experiments performed in this study. Analyses were done in an automated fashion eliminating human bias.

Reporting for specific materials, systems and methods

We require information from authors about some types of materials, experimental systems and methods used in many studies. Here, indicate whether each material, system or method listed is relevant to your study. If you are not sure if a list item applies to your research, read the appropriate section before selecting a response.

Materials & experimental systems

n/a	Involved in the study	
☐	☒	Antibodies
☐	☒	Eukaryotic cell lines
☒	☐	Palaeontology and archaeology
☒	☐	Animals and other organisms
☒	☐	Human research participants
☒	☐	Clinical data
☒	☐	Dual use research of concern

Methods

n/a	Involved in the study	
☐	☒	ChIP-seq
☒	☐	Flow cytometry
☒	☐	MRI-based neuroimaging

Antibodies

Antibodies used	Pol II (Santa Cruz, sc-55492), TBP (Abcam, ab51841), Ser5P (3E8 rat monoclonal obtained from D. Eick, LMU), HA-tag (Cell Signaling Technology, C29F4), and H3K4me3 (Abcam, ab8580) antibodies were used during immunoprecipitations. The amount used in each experiment was specified.
Validation	Pol II (Santa Cruz, sc-55492, https://www.scbt.com/p/pol-ii-antibody-f-12<br>TBP (Abcam, ab51841), https://www.abcam.com/tata-binding-protein-tbp-antibody-mabcam-51841-chip-grade-ab51841.html<br>Ser5P (3E8 rat monoclonal obtained from D. Eick, LMU) is also available from Millipore, https://www.emdmillipore.com/US/en/

product/Anti-RNA-polymerase-II-subunit-B1-phospho-CTD-Ser-5-Antibody-clone-3E8,MM_NF-04-1572
HA-tag (Cell Signaling Technology, C29F4), https://www.cellsignal.com/products/primary-antibodies/ha-tag-c29f4-rabbit-mab/3724
H3K4me3 (Abcam, ab8580), https://www.abcam.com/histone-h3-tri-methyl-k4-antibody-chip-grade-ab8580.html

Eukaryotic cell lines

Policy information about cell lines

Cell line source(s)	HeLa (NCCC)<br><br>MRC5 expressing GFP-tagged Pol II from Steurer, B. et al. Live-cell analysis of endogenous GFP-RPB1 uncovers rapid turnover of initiating and promoter-paused RNA Polymerase II. Proc Natl Acad Sci U S A 115, E4368-E4376 (2018) Provided by Jurgen A Marteijn, Department of Molecular Genetics, Erasmus Medical Center, 3015 AA Rotterdam, The Netherlands; J.Marteijn@erasmusmc.nl<br><br>primary human foreskin fibroblasts (HFF) isolated from de-identified, discarded foreskins<br><br>sf21, Spodoptera frugiperda cells, https://www.thermofisher.com/order/catalog/product/11497013
Authentication	None of the cell lines were authenticated
Mycoplasma contamination	All cell lines were checked for Mycoplasma and tested negative.
Commonly misidentified lines (See ICLAC register)	HeLa cells were used in our study

ChIP-seq

Data deposition

☒ Confirm that both raw and final processed data have been deposited in a public database such as GEO.

☒ Confirm that you have deposited or provided access to graph files (e.g. BED files) for the called peaks.

Data access links *May remain private before publication.*	GSE185763 is publicly available.
Files in database submission	DFF-ChIP:<br><br>GFP-PolIIDFF-Seq8-23-19_20190909000_S1_L004_R1_001.fastq.gz GFP-PolIIDFF-Seq8-23-19_20190909000_S1_L004_R2_001.fastq.gz<br>H3K4me3DFF-Seq8-23-19_20190909000_S2_L004_R1_001.fastq.gz H3K4me3DFF-Seq8-23-19_20190909000_S2_L004_R2_001.fastq.gz<br>PriceBen_1_lane2_20191027000_S16_L002_R1_001.fastq.gz PriceBen_1_lane2_20191027000_S16_L002_R2_001.fastq.gz<br>Price_Ben_2_lane2_20191027000_S13_L002_R1_001.fastq.gz<br>Price_Ben_2_lane2_20191027000_S13_L002_R2_001.fastq.gz<br>Price_Ben_7_lane2_20191027000_S14_L002_R1_001.fastq.gz<br>Price_Ben_7_lane2_20191027000_S14_L002_R2_001.fastq.gz<br>Price_Ben_8_lane2_20191027000_S15_L002_R1_001.fastq.gz<br>Price_Ben_8_lane2_20191027000_S15_L002_R2_001.fastq.gz<br>Sample_13_lane1_20191211000_S5_L001_R1_001.fastq.gz Sample_13_lane1_20191211000_S5_L001_R3_001.fastq.gz<br>Sample_13_1_lane2_20191211000_S21_L002_R1_001.fastq.gz<br>Sample_13_1_lane2_20191211000_S21_L002_R3_001.fastq.gz<br>Sample_15_lane1_20191211000_S7_L001_R1_001.fastq.gz Sample_15_lane1_20191211000_S7_L001_R3_001.fastq.gz<br>Sample_15_1_lane2_20191211000_S23_L002_R1_001.fastq.gz<br>Sample_15_1_lane2_20191211000_S23_L002_R3_001.fastq.gz<br>Sample_14_lane1_20191211000_S6_L001_R1_001.fastq.gz Sample_14_lane1_20191211000_S6_L001_R3_001.fastq.gz<br>Sample_14_1_lane2_20191211000_S22_L002_R1_001.fastq.gz<br>Sample_14_1_lane2_20191211000_S22_L002_R3_001.fastq.gz<br>Sample_16_lane1_20191211000_S8_L001_R1_001.fastq.gz Sample_16_lane1_20191211000_S8_L001_R3_001.fastq.gz<br>Sample_16_1_lane2_20191211000_S24_L002_R1_001.fastq.gz<br>Sample_16_1_lane2_20191211000_S24_L002_R3_001.fastq.gz<br>Sample1_lane1_20200324000_S1_L001_R1_001.fastq.gz Sample1_lane1_20200324000_S1_L001_R3_001.fastq.gz<br>Sample1_lane2_20200324000_S9_L002_R1_001.fastq.gz Sample1_lane2_20200324000_S9_L002_R3_001.fastq.gz<br>Sample2_lane1_20200324000_S2_L001_R1_001.fastq.gz Sample2_lane1_20200324000_S2_L001_R3_001.fastq.gz<br>Sample2_lane2_20200324000_S10_L002_R1_001.fastq.gz Sample2_lane2_20200324000_S10_L002_R3_001.fastq.gz<br>Sample4_lane1_20200324000_S4_L001_R1_001.fastq.gz Sample4_lane1_20200324000_S4_L001_R3_001.fastq.gz<br>Sample4_lane2_20200324000_S12_L002_R1_001.fastq.gz Sample4_lane2_20200324000_S12_L002_R3_001.fastq.gz<br>Sample5_lane1_20200324000_S5_L001_R1_001<br>.fastq.gz Sample5_lane1_20200324000_S5_L001_R3_001.fastq.gz<br>Sample5_lane2_20200324000_S13_L002_R1_001.fastq.gz Sample5_lane2_20200324000_S13_L002_R3_001.fastq.gz<br>Sample6_lane1_20200324000_S6_L001_R1_001.fastq.gz Sample6_lane1_20200324000_S6_L001_R3_001.fastq.gz<br>Sample6_lane2_20200324000_S14_L002_R1_001.fastq.gz Sample6_lane2_20200324000_S14_L002_R3_001.fastq.gz<br>Sample7_lane1_20200324000_S7_L001_R1_001.fastq.gz Sample7_lane1_20200324000_S7_L001_R3_001.fastq.gz

Sample7_lane2_20200324000_S15_L002_R1_001.fastq.gz Sample7_lane2_20200324000_S15_L002_R3_001.fastq.gz
Sample1_lane1_20200930000_S1_L001_R1_001.fastq.gz Sample1_lane1_20200930000_S1_L001_R3_001.fastq.gz
Sample1_lane2_20200930000_S13_L002_R1_001.fastq.gz Sample1_lane2_20200930000_S13_L002_R3_001.fastq.gz
Sample5_lane1_20200930000_S8_L001_R1_001.fastq.gz Sample5_lane1_20200930000_S8_L001_R3_001.fastq.gz
Sample5_lane2_20200930000_S20_L002_R1_001.fastq.gz Sample5_lane2_20200930000_S20_L002_R3_001.fastq.gz
Sample4_lane1_20200930000_S7_L001_R1_001.fastq.gz Sample4_lane1_20200930000_S7_L001_R3_001.fastq.gz
Sample4_lane2_20200930000_S19_L002_R1_001.fastq.gz Sample4_lane2_20200930000_S19_L002_R3_001.fastq.gz
Sample8_lane1_20200930000_S11_L001_R1_001.fastq.gz Sample8_lane1_20200930000_S11_L001_R3_001.fastq.gz
Sample8_lane2_20200930000_S23_L002_R1_001.fastq.gz Sample8_lane2_20200930000_S23_L002_R3_001.fastq.gz
Sample7_lane1_20200930000_S10_L001_R1_001.fastq.gz Sample7_lane1_20200930000_S10_L001_R3_001.fastq.gz
Exp1_MRC5_Flavo_Pol-II_hg38.bw
Exp1_HeLa_Flavo_H3K4me3_hg38.bw
Exp2_MRC5_H3K4me3_hg38.bw
Exp2_MRC5_Flavo_H3K4me3_hg38.bw
Exp2_MRC5_HS_Pol-II_hg38.bw
Exp2_MRC5_Flavo-HS_Pol-II_hg38.bw
Exp3_HFF_H3K4me3_hg38.bw
Exp3_HFF_Pol-II_hg38.bw
Exp3_HFF_TB40e_H3K4me3_hg38.bw
Exp3_HFF_TB40e_Pol-II_hg38.bw
Exp4_HFF_TB40e_Pol-II_hg38.bw
Exp4_HFF_TB40e_Trp_Pol-II_hg38.bw
Exp4_HFF_TB40e_TBP_hg38.bw
Exp4_HFF_TB40e_Ser5P_hg38.bw
Exp4_HFF_Towne_UL87_hg38.bw
Exp4_HFF_TB40e_H3K4me3_hg38.bw
Exp5_HFF_Towne_Pol-II_Rep1_hg38.bw
Exp5_HFF_Towne_Pol-II_Rep2_hg38.bw
Exp5_HFF_Towne_Ser5P_Rep1_hg38.bw
Exp5_HFF_Towne_Ser5P_Rep2_hg38.bw
Exp5_HFF_Towne_TBP_Rep1_hg38.bw
Exp5_HFF_Towne_TBP_Rep2_hg38.bw
Exp5_HFF_Towne_H3K4me3_Rep1_hg38.bw
Exp5_HFF_Towne_H3K4me3_Rep2_hg38.bw
Exp5_HFF_Towne_UL87_Rep1_hg38.bw
Exp3_HFF_TB40e_H3K4me3_KF297339.1.bw
Exp3_HFF_TB40e_Pol-II_KF297339.1.bw
Exp4_HFF_TB40e_Pol-II_KF297339.1.bw
Exp4_HFF_TB40e_Trp_Pol-II_KF297339.1.bw
Exp4_HFF_TB40e_TBP_KF297339.1.bw
Exp4_HFF_TB40e_Ser5P_KF297339.1.bw
Exp4_HFF_Towne_UL87_FJ616285.1.bw
Exp4_HFF_TB40e_UL87_KF297339.1.bw
Exp4_HFF_TB40e_H3K4me3_KF297339.1.bw
Exp5_HFF_Towne_Pol-II_Rep1_FJ616285.1.bw
Exp5_HFF_Towne_Pol-II_Rep2_FJ616285.1.bw
Exp5_HFF_Towne_Ser5P_Rep1_FJ616285.1.bw
Exp5_HFF_Towne_Ser5P_Rep2_FJ616285.1.bw
Exp5_HFF_Towne_TBP_Rep1_FJ616285.1.bw
Exp5_HFF_Towne_TBP_Rep2_FJ616285.1.bw
Exp5_HFF_Towne_H3K4me3_Rep1_FJ616285.1.bw
Exp5_HFF_Towne_H3K4me3_Rep2_FJ616285.1.bw
Exp5_HFF_Towne_UL87_Rep1_FJ616285.1.bw

PRO-Seq:

Sample1_20180807000_S13_L004_R1_001.fastq.gz
Sample1_20180807000_S13_L004_R2_001.fastq.gz
Sample1_20180807001_S21_L005_R1_001.fastq.gz
Sample1_20180807001_S21_L005_R2_001.fastq.gz
Sample2_20180807000_S14_L004_R1_001.fastq.gz
Sample2_20180807000_S14_L004_R2_001.fastq.gz
Sample3_20180807000_S15_L004_R1_001.fastq.gz
Sample3_20180807000_S15_L004_R2_001.fastq.gz
Sample4_20180807000_S16_L004_R1_001.fastq.gz
Sample4_20180807000_S16_L004_R2_001.fastq.gz
Sample5_20180807000_S17_L004_R1_001.fastq.gz
Sample5_20180807000_S17_L004_R2_001.fastq.gz
Sample6_20180807000_S18_L004_R1_001.fastq.gz
Sample6_20180807000_S18_L004_R2_001.fastq.gz
Sample7_20180807000_S19_L004_R1_001.fastq.gz
Sample7_20180807000_S19_L004_R2_001.fastq.gz
Sample8_20180807000_S20_L004_R1_001.fastq.gz
Sample8_20180807000_S20_L004_R2_001.fastq.gz
Sample2_20180807001_S22_L005_R1_001.fastq.gz
Sample2_20180807001_S22_L005_R2_001.fastq.gz

Sample3_20180807001_S23_L005_R1_001.fastq.gz
Sample3_20180807001_S23_L005_R2_001.fastq.gz
Sample4_20180807001_S24_L005_R1_001.fastq.gz
Sample4_20180807001_S24_L005_R2_001.fastq.gz
Sample5_20180807001_S25_L005_R1_001.fastq.gz
Sample5_20180807001_S25_L005_R2_001.fastq.gz
Sample6_20180807001_S26_L005_R1_001.fastq.gz
Sample6_20180807001_S26_L005_R2_001.fastq.gz
22018-08-07-HFF-hg38-SIC-FW.bw
2018-08-07-HFF-hg38-SIC-RV.bw
2018-08-07-HFF-KF297339.1-SIC-FW.bw
2018-08-07-HFF-KF297339.1-SIC-RV.bw
2018-08-07-HFF-Flavo-hg38-SIC-FW.bw
2018-08-07-HFF-Flavo-hg38-SIC-RV.bw
2018-08-07-HFF-Flavo-KF297339.1-SIC-FW.bw
2018-08-07-HFF-Flavo-KF297339.1-SIC-RV.bw
2018-08-07-HFF-Flavo-combined-dedup.HG38.5prime.FW.SIC.bw
2018-08-07-HFF-Flavo-combined-dedup.hg38.5prime.RV.SIC.bw
2018-08-07-HFF-Flavo-combined-dedup.KF297339.1.5prime.FW.SIC.bw
2018-08-07-HFF-Flavo-combined-dedup.KF297339.1.5prime.RV.SIC.bw
2018-08-07-HFF-TB40e-4hpi-hg38-SIC-FW.bw
2018-08-07-HFF-TB40e-4hpi-hg38-SIC-RV.bw
2018-08-07-HFF-TB40e-4hpi-KF297339.1-SIC-FW.bw
2018-08-07-HFF-TB40e-4hpi-KF297339.1-SIC-RV.bw
2018-08-07-HFF-TB40e-4hpi-Flavo-hg38-SIC-FW.bw
2018-08-07-HFF-TB40e-4hpi-Flavo-hg38-SIC-RV.bw
2018-08-07-HFF-TB40e-4hpi-Flavo-KF297339.1-SIC-FW.bw
2018-08-07-HFF-TB40e-4hpi-Flavo-KF297339.1-SIC-RV.bw
2018-08-07-HFF-TB40e-4hpi-Flavo-combined-dedup.hg38.5prime.FW.SIC.bw
2018-08-07-HFF-TB40e-4hpi-Flavo-combined-dedup.hg38.5prime.RV.SIC.bw
2018-08-07-HFF-TB40e-4hpi-Flavo-combined-dedup.KF297339.1.5prime.FW.SIC.bw
2018-08-07-HFF-TB40e-4hpi-Flavo-combined-dedup.KF297339.1.5prime.RV.SIC.bw
2018-08-07-HFF-TB40e-12hpi-hg38-SIC-FW.bw
2018-08-07-HFF-TB40e-12hpi-hg38-SIC-RV.bw
2018-08-07-HFF-TB40e-12hpi-KF297339.1-SIC-FW.bw
2018-08-07-HFF-TB40e-12hpi-KF297339.1-SIC-RV.bw
2018-08-07-HFF-TB40e-12hpi-Flavo-hg38-SIC-FW.bw
2018-08-07-HFF-TB40e-12hpi-Flavo-hg38-SIC-RV.bw
2018-08-07-HFF-TB40e-12hpi-Flavo-KF297339.1-SIC-FW.bw
2018-08-07-HFF-TB40e-12hpi-Flavo-KF297339.1-SIC-RV.bw
2018-08-07-HFF-TB40e-12hpi-Flavo-combined-dedup.hg38.5prime.FW.SIC.bw
2018-08-07-HFF-TB40e-12hpi-Flavo-combined-dedup.hg38.5prime.RV.SIC.bw
2018-08-07-HFF-TB40e-12hpi-Flavo-combined-dedup.KF297339.1.5prime.FW.SIC.bw
2018-08-07-HFF-TB40e-12hpi-Flavo-combined-dedup.KF297339.1.5prime.RV.SIC.bw
2018-08-07-HFF-TB40e-24hpi-hg38-SIC-FW.bw
2018-08-07-HFF-TB40e-24hpi-hg38-SIC-RV.bw
2018-08-07-HFF-TB40e-24hpi-KF297339.1-SIC-FW.bw
2018-08-07-HFF-TB40e-24hpi-KF297339.1-SIC-RV.bw
2018-08-07-HFF-TB40e-24hpi-Flavo-hg38-SIC-FW.bw
2018-08-07-HFF-TB40e-24hpi-Flavo-hg38-SIC-RV.bw
2018-08-07-HFF-TB40e-24hpi-Flavo-KF297339.1-SIC-FW.bw
2018-08-07-HFF-TB40e-24hpi-Flavo-KF297339.1-SIC-RV.bw
2018-08-07-HFF-TB40e-24hpi-Flavo-combined-dedup.hg38.5prime.FW.SIC.bw
2018-08-07-HFF-TB40e-24hpi-Flavo-combined-dedup.hg38.5prime.RV.SIC.bw
2018-08-07-HFF-TB40e-24hpi-Flavo-combined-dedup.KF297339.1.5prime.FW.SIC.bw
2018-08-07-HFF-TB40e-24hpi-Flavo-combined-dedup.KF297339.1.5prime.RV.SIC.bw
2018-08-07-HFF-TB40e-48hpi-hg38-SIC-FW.bw
2018-08-07-HFF-TB40e-48hpi-hg38-SIC-RV.bw
2018-08-07-HFF-TB40e-48hpi-KF297339.1-SIC-FW.bw
2018-08-07-HFF-TB40e-48hpi-KF297339.1-SIC-RV.bw
2018-08-07-HFF-TB40e-48hpi-Flavo-hg38-SIC-FW.bw
2018-08-07-HFF-TB40e-48hpi-Flavo-hg38-SIC-RV.bw
2018-08-07-HFF-TB40e-48hpi-Flavo-KF297339.1-SIC-FW.bw
2018-08-07-HFF-TB40e-48hpi-Flavo-KF297339.1-SIC-RV.bw
2018-08-07-HFF-TB40e-48hpi-Flavo-combined-dedup.hg38.5prime.FW.SIC.bw
2018-08-07-HFF-TB40e-48hpi-Flavo-combined-dedup.hg38.5prime.RV.SIC.bw
2018-08-07-HFF-TB40e-48hpi-Flavo-combined-dedup.KF297339.1.5prime.FW.SIC.bw
2018-08-07-HFF-TB40e-48hpi-Flavo-combined-dedup.KF297339.1.5prime.RV.SIC.bw
2018-08-07-HFF-TB40e-72hpi-hg38-SIC-FW.bw
2018-08-07-HFF-TB40e-72hpi-hg38-SIC-RV.bw
2018-08-07-HFF-TB40e-72hpi-KF297339.1-SIC-FW.bw
2018-08-07-HFF-TB40e-72hpi-KF297339.1-SIC-RV.bw
2018-08-07-HFF-TB40e-72hpi-Flavo-hg38-SIC-FW.bw
2018-08-07-HFF-TB40e-72hpi-Flavo-hg38-SIC-RV.bw
2018-08-07-HFF-TB40e-72hpi-Flavo-KF297339.1-SIC-FW.bw
2018-08-07-HFF-TB40e-72hpi-Flavo-KF297339.1-SIC-RV.bw

2018-08-07-HFF-TB40e-72hpi-Flavo-combined-dedup.hg38.5prime.FW.SIC.bw
2018-08-07-HFF-TB40e-72hpi-Flavo-combined-dedup.hg38.5prime.RV.SIC.bw
2018-08-07-HFF-TB40e-72hpi-Flavo-combined-dedup.KF297339.1.5prime.FW.SIC.bw
2018-08-07-HFF-TB40e-72hpi-Flavo-combined-dedup.KF297339.1.5prime.RV.SIC.bw
2018-08-07-PRO-Cap-HFF-TB40e-72hpi-KF297339.1.FW.bw
2018-08-07-PRO-Cap-HFF-TB40e-72hpi-KF297339.1.RV.bw
2018-08-07-PRO-Cap-HFF-TB40e-72hpi-dedup-KF297339.1_5prime-FW.bw
2018-08-07-PRO-Cap-HFF-TB40e-72hpi-dedup-KF297339.1_5prime-RV.bw

Genome browser session
(e.g. UCSC)

HOST: https://genome.ucsc.edu/s/David%20Price/Host
Towne: https://genome.ucsc.edu/s/David%20Price/Towne
TB40/E: https://genome.ucsc.edu/s/David%20Price/TB40_E

Methodology

Replicates

At least 2 replicas for PRO-Seq and DFF-ChIP datasets were performed. Many correlations were performed and are part of the figures. When possible experiments were carried out at separate times including on different cell types as described earlier.

Sequencing depth

Cell line Treatment IP Sample # Total frags No UMIs Deduped frags UMI hg38 hg38 DMSO-treated TSRs TB40e TB40e 72 hpi DMSO-treated TSRs Towne Towne 96 hpi DMSO-treated TSRs Ratio virus/hg38 reads
DFF-seq Experiment 0
HeLa Flavo NA 0 259,878,094 NA 259,878,094 NasCap NA   NA   NA   NA
DFF-ChIP Experiment 1
MRC5 Flavo Low Salt GFP-Pol II 1 154,115,887 NA 154,115,887 PRO-Cap NA   NA   NA   NA
HeLa Flavo Low Salt H3K4me3 2 134,346,538 NA 134,346,538 NasCap NA   NA   NA   NA
DFF-ChIP Experiment 2
MRC5 Control Low Salt H3K4me3 1 72,834,422 NA 72,834,422 PRO-Cap NA   NA   NA   NA
MRC5 Flavo Low Salt H3K4me3 2 66,443,675 NA 66,443,675 PRO-Cap NA   NA   NA   NA
MRC5 Control High Salt GFP-Pol II 7 79,232,994 NA 79,232,994 PRO-Cap NA   NA   NA   NA
MRC5 Flavo High Salt GFP-Pol II 8 74,809,416 NA 74,809,416 PRO-Cap NA   NA   NA   NA
DFF-ChIP Experiment 3
HFF Uninfected Low Salt H3K4me3 13 NA 134,011,231 133,996,789 PRO-Cap 14,442 PRO-Cap   NA   NA 0.000
HFF Uninfected Low Salt Pol II 15 NA 157,201,638 157,187,105 PRO-Cap 14,533 PRO-Cap   NA   NA 0.000
HFF 48 hrs TB40e infected Low Salt H3K4me3 14 NA 155,731,479 148,129,377 PRO-Cap 7,602,102 PRO-Cap   NA   NA 0.051
HFF 48 hrs TB40e infected Low Salt Pol II 16 NA 162,632,770 153,472,304 PRO-Cap 9,160,466 PRO-Cap   NA   NA 0.060
DFF-ChIP Experiment 4
HFF 48 hrs TB40e infected Low Salt Pol II 1 NA 70,551,289 67,131,252 PRO-Cap 3,420,037 PRO-Cap   NA   NA 0.051
HFF 48 hrs TB40e infected Low Salt Pol II triptolide 2 NA 71,665,592 71,163,546 PRO-Cap 502,046 PRO-Cap   NA   NA 0.007
HFF 48 hrs TB40e infected Low Salt TBP 4 NA 77,257,554 75,331,297 PRO-Cap 1,926,257 PRO-Cap   NA   NA 0.026
HFF 48 hrs TB40e infected Low Salt Pol II Ser5P 5 NA 60,509,273 59,744,474 PRO-Cap 764,799 PRO-Cap   NA   NA 0.013
HFF 48 hrs Towne infected Low Salt UL87 6 NA 64,484,001 61,762,335 PRO-Cap 2,721,666 PRO-Cap 3,708,383 PRO-Cap 0.044
HFF 48 hrs TB40e infected Low Salt H3K4me3 7 NA 73,447,814 72,405,453 PRO-Cap 1,042,361 PRO-Cap   NA   NA 0.014
HFF 48 hrs TB40e infected Low Salt H3K36me3 8 NA 68,279,161 68,122,582 PRO-Cap 156,579 PRO-Cap   NA   NA 0.002
DFF-ChIP Experiment 5
HFF 48 hrs Towne infected Low Salt Pol II Rep1 1 NA 27,966,122 27,793,597 PRO-Cap NA   NA 172,525 PRO-Cap 0.006
HFF 48 hrs Towne infected Low Salt Pol II Rep2 1 NA 23,502,831 23,376,624 PRO-Cap NA   NA 126,207 PRO-Cap 0.005
HFF 48 hrs Towne infected Low Salt Ser5P Rep1 5 NA 36,939,096 36,817,587 PRO-Cap NA   NA 121,509 PRO-Cap 0.003
HFF 48 hrs Towne infected Low Salt Ser5P Rep2 5 NA 32,851,173 32,794,225 PRO-Cap NA   NA 56,948 PRO-Cap 0.002
HFF 48 hrs Towne infected Low Salt TBP Rep1 4 NA 27,037,976 26,932,769 PRO-Cap NA   NA 105,207 PRO-Cap 0.004
HFF 48 hrs Towne infected Low Salt TBP Rep2 4 NA 18,313,058 18,281,156 PRO-Cap NA   NA 31,902 PRO-Cap 0.002
HFF 48 hrs Towne infected Low Salt H3K4me3 Rep1 8 NA 30,432,567 30,394,222 PRO-Cap NA   NA 38,345 PRO-Cap 0.001
HFF 48 hrs Towne infected Low Salt H3K4me3 Rep2 8 NA 33,531,259 33,496,916 PRO-Cap NA   NA 34,343 PRO-Cap 0.001
HFF 48 hrs Towne infected Low Salt UL87 Rep1 7 NA 18,362,586 18,157,172 PRO-Cap NA   NA 205,414 PRO-Cap 0.011

Antibodies

Pol II (Santa Cruz, sc-55492, https://www.scbt.com/p/pol-ii-antibody-f-12
TBP (Abcam, ab51841), https://www.abcam.com/tata-binding-protein-tbp-antibody-mabcam-51841-chip-grade-ab51841.html
Ser5P (Millipore, 3E8), https://www.emdmillipore.com/US/en/product/Anti-RNA-polymerase-II-subunit-B1-phospho-CTD-Ser-5-Antibody-clone-3E8,MM_NF-04-1572
HA-tag (Cell Signaling Technology, C29F4), https://www.cellsignal.com/products/primary-antibodies/ha-tag-c29f4-rabbit-mab/3724
H3K4me3 (Abcam, ab8580), https://www.abcam.com/histone-h3-tri-methyl-k4-antibody-chip-grade-ab8580.html

Peak calling parameters

We did not use peak calling

Data quality

Data was of extremely high quality as evidenced by the genome browser tracks provided

Software

Custom Software used:
DNAfastqtoBigWig (https://github.com/P-TEFb/DNAfastqtoBigWig)
ABD.py (https://github.com/P-TEFb/ABD)
fragMap.py (https://github.com/P-TEFb/fragMap)

Initial workup of data was performed using DNAfastqtoBigWig (https://github.com/P-TEFb/DNAfastqtoBigWig). This is a linux based, multi-thread capable, Next Generation Sequencing (NGS) data analysis program with a command line interface. It performs the standard NGS data processing steps including, downloading sequencing data from a given web server, trimming adapter sequences from the sequencing data, aligning the trimmed data to a given list of genomes, generating mapping statistics for the aligned data, deduplication of aligned fragments using their Unique Molecular Identifiers (UMI), and finally, generating tracks for each sample in bigwig format. The program automatically accomplishes the following steps. Raw sequences in Fastq format were downloaded from the Iowa Institute of Human Genetics (IIHG) Genome Sequencing web server using wget command. Next, adapter sequences were trimmed from these sequences using trim_galore 0.6 (https://github.com/FelixKrueger/TrimGalore/releases/tag/0.6.6) while retaining only paired end trimmed sequences of at least 18 bp in size. These sequences were aligned with UCSC hg38, and Genbank TB40/E and Towne HCMV assemblies using bowtie v1.2.2 to generate alignments in sam format. UMIs reads were used to deduplicate the aligned reads which were then converted into bed files. Unstranded tracks were generated for each sample by first converting bed into bedGraph format using bedtools v2.26, and subsequently into bigwig format using the Kent UCSC utility program called bedGraphToBigWig. All datasets are described in the Supplementary Data file. Raw and processed sequencing data can be obtained from GEO (GSE185763).

Fragment distribution plots
Fragment size frequencies were calculated by counting the number of times a single fragment size or a range of fragment sizes are present in a given sample or overlap to a list of genomic intervals of a specific size. Bedtools v2.26 intersect program was used to generate overlap data between fragment and genomic intervals. Bash and awk scripts were used to generate counts for a single or a range of fragment sizes. Next, fragment sizes and their associated counts were sorted from short to long order. Additionally, fragment size counts were normalized to generate their relative amounts by dividing them with the total number of fragments present in a sample. MS Excel was used to plot relative and absolute counts of fragment sizes for each sample using scatter with straight lines option.

Average base distribution plots
The number of times a nucleotide is present at each position within a specified genomic interval was calculated as follows. Bedtools v2.26 getfasta program was used to generate FASTA files while maintaining same strand orientation for ±100 bp genomic intervals centered on fragment starts and ends. For these analyses all intervals centered on fragment starts were marked positive strand and intervals centered on fragment ends were marked negative strand. A custom linux based, multi-thread capable python script called ABD.py (https://github.com/P-TEFb/ABD) was run to generate absolute counts for each nucleotide across the given genomic intervals. Absolute nucleotide counts were converted to fractions by dividing them with the total number of sequences. MS Excel was used to plot base fractions across the genomic interval using scatter with straight lines option. Finally, the following color code was used for these plots: A was blue (hex code #2222ff), T was orange (hex code #ff6600), G was gray (hex code #bbbbbb), and C was yellow (hex code #dddd00).

MaxTSSs analysis
The truQuant program28 with an updated list of black listed regions was run on the PRO-Cap data (GSE113394)25 generated from uninfected HFF cells and DMSO bound NAS-Cap data (GSE139237)6 generated from HeLa cells using published parameters to generate host specific MaxTSSs. 12,229 (PRO-Cap) and 12,201 (NasCap) MaxTSSs associated with known host genes were used for further analysis. TsrFinderM26 was run on PRO-Cap datasets from TB40/E infected HFFs 72 hpi (GSE185763) and Towne infected HFFs 96 hpi (GSE113394) using published parameters to generate HMCV specific MaxTSSs. MaxTSSs associated with RNA 4.9 and without a ±1000 bp genomic sequence were excluded from further analysis. 1,461 TB40/E and 1,456 Towne MaxTSSs were used for further analysis.
